# Added Values of Time Series in Material Flow Analysis: The Austrian Phosphorus Budget from 1990 to 2011

**DOI:** 10.1111/jiec.12381

**Published:** 2015-12-22

**Authors:** Ottavia Zoboli, David Laner, Matthias Zessner, Helmut Rechberger

**Affiliations:** Center for Water Resource Systems, TU Wien, Karlsplatz 13/222, A-1040 Vienna, Austria

**Keywords:** data reconciliation, industrial ecology, phosphorus, substance flow analysis (SFA), time series, uncertainty

## Abstract

Material flow analysis is a tool that is increasingly used as a foundation for resource management and environmental protection. This tool is primarily applied in a static manner to individual years, ignoring the impact of time on the material budgets. In this study, a detailed multiyear model of the Austrian phosphorus budget covering the period 1990–2011 was built to investigate its behavior over time and test the hypothesis that a multiyear approach can also contribute to the improvement of static budgets. Further, a novel method was applied to investigate the quality and characteristics of the data and quantify the uncertainty. The degree of change between the budgets was assessed and showed that approximately half of the flows have changed significantly and, at times, abruptly since 1990, but it is not possible to distinguish unequivocally between constant and moderately changing flows given their uncertainty. The study reveals that the phosphorus transported in waste flows has increased more rapidly than its recovery, which accounted for 55% to 60% of the total waste phosphorus in 1990 and only 40% in 2011. The loss ratio in landfills and cement kilns has oscillated in the range of 40% to 50%. From a methodological point of view, the multiyear approach has broadened the conceptual model of the budget, making it more suitable as a basis for material accounting and monitoring. Moreover, the analysis of the data reconciliation process over a long period of time proved to be a useful tool for identifying systematic errors in the model.

**Supplementary Information:**

The online version of this article (doi:10.1111/jiec.12381) contains supplementary material, which is available to authorized users.

## Introduction

### Importance and Challenges of Phosphorus

The role played by phosphorus (P) in modern societies is essential and irreplaceable. Not only is it often a limiting factor for growth and a fundamental component of fertilizers, but it also finds manifold industrial applications, such as in car engine lubricants, pesticides, rechargeable batteries, and flame retardants (Schipper 2014). P is exclusively obtained from mineral deposits, and its future availability is currently plagued by uncertainty. The figures for phosphate rock reserves and resources as well as the models applied to estimate their peak production and life span are still highly debated, with predictions ranging from a few decades to hundreds of years (Cordell et al. 2009; Van Kauwenbergh 2010; Van Vuuren et al. 2010; Cooper et al. 2011; Cordell and White 2011; Sverdrup and Ragnarsdottir 2011; Vaccari and Strigul 2011; Ott and Rechberger 2012; Edixhoven et al. 2013; Koppelaar and Weikard 2013; Mohr 2013; Scholz and Wellmer 2013). Notwithstanding the disputed scarcity concern, there are other important rationales to pursuing a more efficient and sustainable P management. The most pressing issues are water body eutrophication, accumulation of heavy metals in agricultural soils through the application of mineral fertilizers, and geopolitical and equity risks associated with the concentration of mineral deposits in a handful of countries (Condron et al. 2013; Ulrich et al. 2013a, 2013b). Based on the supply risk, the European Commission has recently added phosphate rock to the revised list of critical raw materials (European Commission 2013).

### One-Year and Historical Phosphorus Material Flow Analysis

As shown by Ulrich and Schnug (2013), since 2008 multistakeholder platforms have been formed and researchers have intensified their effort in tackling the challenges related to P. Material flow analysis (MFA) has played a key role in assessing flows and stocks and identifying hotspots of poor management and recycling potential, as manifested by two recent reviews of P MFAs carried out at different scales throughout the world (Cordell et al. 2012; Chowdhury et al. 2014). Until recently, these studies have primarily focused on static 1-year budgets (balanced models of material flows and stocks obtained through MFA methodology), and the review carried out by Chowdhury and colleagues (2014) identified the paucity of multiyear analyses as a major knowledge gap. The few efforts put forth in this direction have led to significant outcomes.

Li and colleagues (2012) analyzed the urban P metabolism through food consumption in China from 1986 to 2006 and identified the relationships among urban P inflow and population growth, dietary changes, and velocity of urban area expansion. Further, their research showed how the rising urban per capita income has altered the urban P stock and the P outflow to surrounding nonurban ecosystems. Ma and colleagues (2012) conducted a P MFA for all of China from 1984 to 2008, which not only showed a significant increase in P consumption and waste, an increasing (though fluctuating) P ore extraction, and a decline in P recycling rates, but also highlighted the correlation between these changes and specific socioeconomic factors. Although European countries have not undergone socioeconomic changes as drastic as those of China in recent decades, Senthilkumar and colleagues (2012) revealed that France halved the use of mineral fertilizers in agriculture from 1990 to 2006, while maintaining the same levels of agricultural production and losses to the environment. Neset and colleagues (2008) analyzed the flow of P in food consumption and production for a city in Sweden in the period 1870–2000 and found an increase in P reaching consumers, owing both to population growth and to a shift toward a diet richer in meat and dairy products. Their study also analyzed the related outflow of P in waste and wastewater, revealing a steep decline in the ratio between reuse and losses. Lamprecht and colleagues (2011) quantified the high impact that the Bovine Spongiform Encephalopathy crisis exerted on the P cycle in Switzerland by banning the reuse of P-rich meat and bone meal as animal feed.

These examples prove that multiyear MFAs can provide insights with important implications for environmental, resource, and waste management. In economy-wide accounting and analysis, multiyear schemes are already widespread to the extent that several countries have incorporated them into their statistical information systems (Fischer-Kowalski et al. 2011). As suggested by Chowdhury and colleagues (2014), however, the main obstacle that might dissuade most researchers from conducting multiyear studies in the specific field of phosphorus is the lack of data. In this respect, Austria offers a favorable case study owing to the existence of a detailed budget representing an average year between 2004 and 2008 (Egle et al. 2014) and large data availability for the past.

This study presents a multiyear analysis of the Austrian P budget covering the period from 1990 to 2011. The first aim of the work is to identify and assess the extent of the temporal changes that occurred in the system during the last two decades. It differs from previous multiyear P MFAs owing to the very high level of detail of the model, which allows for an unprecedented systematic assessment. One of the main novelties lies in the characterization of data quality and uncertainty, as well as in the quantification and discussion of the implications of the latter for the assessment of temporal changes. The second goal of this contribution is to test the hypothesis that a multiyear MFA can lead to a better understanding of the data quality and to the improvement of the model itself, which, in turn, would benefit the 1-year static budgets. Therefore, although investigating the changing traits of P flows and stocks is relevant per se, this work tests novel methodological approaches that could prove useful in the study of other materials.

### Data Uncertainty

Although the uncertainty of the data has been increasingly recognized as an essential aspect in MFA studies, in reality it is still often ignored or only qualitatively discussed (Laner et al. 2014; Rechberger et al. 2014). As presented in the review by Laner and colleagues (2014), there exist different approaches that can be grouped as follows: (1) qualitative and semiquantitative methods, that reflect the authors’ confidence in the results without formally assessing the quality of the data; (2) methods based on data classification, used to evaluate the data quality without applying rigorous mathematical procedures to propagate uncertainties in the model; and (3) statistical methods, which do propagate uncertainties in the model. This study applies a novel approach developed by Laner and colleagues (2015), which builds on the idea proposed by Hedbrandt and Sörme (2001) of quantifying the material flow uncertainty combining data classification and exponential-type uncertainty characterization functions, and on the scheme introduced by Weidema and Wesnæs (1996) to assess the quality of life cycle inventory data. This novel approach was selected for different reasons. In the first place, it is based on a characterization of the data quality through the use of different indicators, which allows describing the characteristics of the data and identifying specific problems. Second, the quantification of the uncertainty follows a procedure designed to ensure its consistency within the model. Moreover, it is based on coefficients of variation, making the results directly utilizable for the MFA software STAN applied to balance the model in this study. Last, this method was chosen in order to provide a well-documented and transparent characterization of the uncertainty.

## Materials and Methods

### Model and Data

This work follows the methodology of MFA described by Brunner and Rechberger (2004) and addresses the flows of P on the national level. According to the classification suggested by Van der Voet (2002), this work belongs to the *Accounting* category, which represents the most suitable modeling approach for keeping track of flows and stocks in order to enable policy makers to detect trends and assess the performance of implemented measures, and also for identifying missing or inaccurate data. The design consists of a multiyear static model, which simultaneously balances 22 time nodes (yearly snapshots). The 22 time nodes are independent, in that data-based values for the flows are introduced as input to the system for each one of the studied years. The only elements that are not independent among time nodes are stocks, for which only the initial value for the year 1990 is introduced as input, whereas thereafter their total amount is obtained by calculating the stock changes in each individual year based on closed mass balances. The reason behind this choice is that only poor information is available to calculate the total value of stocks, so that it is preferable to carry out such estimations only as a starting point and let the rest be based on the flows, for which better data are available. Although stocks are not entirely time independent, this approach differs substantially from MFA dynamic modeling, which includes time as a modeling parameter to explore the behavior of stocks and flows in time. Dynamic modeling is mostly applied for the analysis of accumulated stocks of metals and other persistent toxics and to predict future scenarios with respect to waste flows and emissions (Van der Voet 2002; Müller et al. 2014). The multiyear static model presented here does not capture the dynamics between stocks and future flows and therefore does not aim at predicting future scenarios. It is instead designed to perform a descriptive, detailed, and data-based analysis of the system over time.

**Figure 1:**
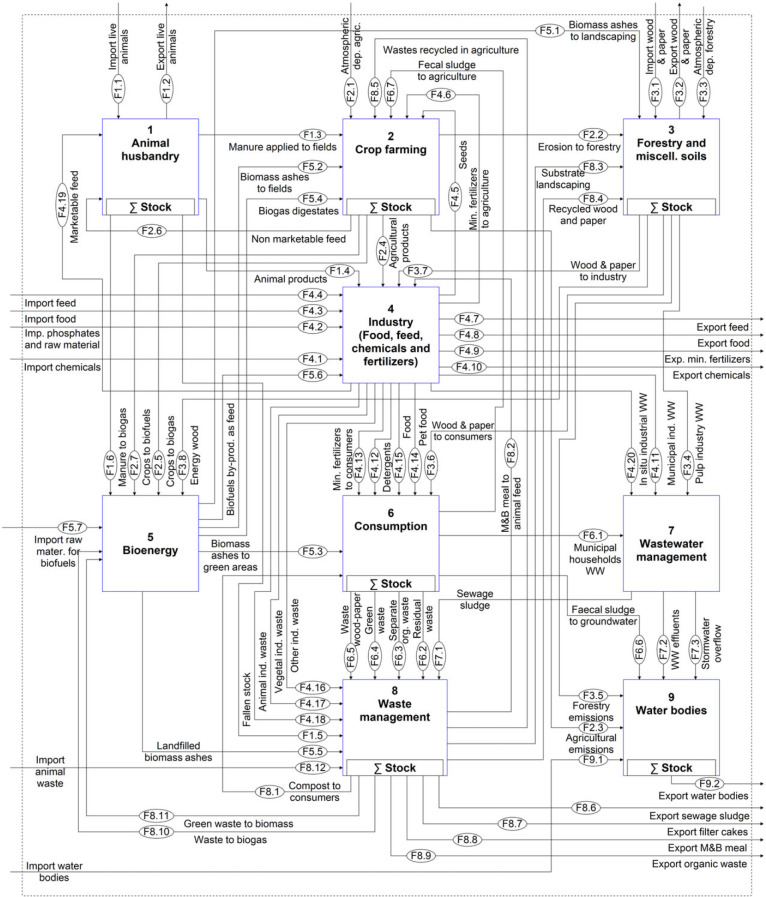
Qualitative MFA model of the Austrian P budget. MFA = material flow analysis; P = phosphorus; F values in the ovals indicate the number assigned to each flow.

The work of Egle and colleagues (2014) is used as a foundation to conceptually describe the national P budget. Given that the model structure is identical for the whole time series, some modifications had to be made to the existing model. A flow of meat and bone meal used as animal feed was added, because it represented a very important use before the ban introduced in the year 2001. Further, the recent expansion of the bioenergy sector justifies this process being highlighted separately. In contrast, the results of Egle and colleagues (2014) revealed that the role of the chemical industry in Austria is not so significant that it requires a separate process; therefore, it was lumped within the *Industry* process, which also encompasses the trade and production of food, feed, raw phosphates, and fertilizers. The adjusted model is shown in figure 1 and is structured in nine processes that, with the exception of *Crop farming* and *Water bodies*, are described by subsystems, which are available as Supporting Information available on the Journal's website (S1). In total, the system is composed of 56 processes, eight stocks, 122 flows, and two transfer coefficients.

With respect to the input values, a detailed description of the equations and data sources used in the calculations is available as Supporting Information on the Web (S2). The majority of the flows were obtained by multiplying goods (defined as substances or mixtures of substances that have economic values assigned by markets, according to Brunner and Rechberger [2004]) with their P concentration. For a subset of flows, however, data on the P flows (P mass per time) were directly available given the abundance of research and statistical accounting already developed for this substance. Examples of this occurrence are wastewater, sewage sludge and effluents, mineral fertilizers, and P flows in the environment, namely, atmospheric deposition and emissions to water bodies from agricultural and forestry soils. For the goods and the direct P flows, all of the compartments of the system are characterized by large data availability, but they do show important differences with respect to the frequency and level of detail. For the production, import, export, and consumption of food and animal feed products, highly detailed and yearly data are available in the supply balance sheets. National statistics and official sector reports also provide further detailed and yearly data for the processes *Animal husbandry*, *Crop farming*, and *Forestry*; for food consumption; and for the import/export of chemicals. With respect to the *Bioenergy* process, it is necessary to make a distinction between its subprocesses. Biofuels production is highly regulated and documented in official yearly reports, although attention is mostly focused on the biofuels themselves and not as much on the materials used for their production or on their by-products, which is the relevant information for the P budget. The data on biogas or biomass plants, in contrast, offer more information on input materials and by-products, but are not available with the same regularity. In compliance with the European Directive 91/271/EEC on urban wastewater treatment, official reports (published with a frequency that recently increased to biannual) provide data on the P loads in and out of municipal treatment plants as well as information on the average P removal rate and the connection rate to the sewage system. Direct data on the specific contribution of households and industries to the total P load are not available, and therefore these quantities were estimated based on information on the average P load per capita. The most important source for *Waste management* is represented by the national waste management plans, which contain information on the volumes of wastes generated, their main origins, management, and disposal. Such plans are not a satisfactory source for this study, because they are published only every 5 years and also because they often present a high level of aggregation, especially for the management and disposal of waste flows. This information could be partially complemented with official reports that provide greater detail on individual processes or specific waste flows, certain of which also supply multiyear information. This notwithstanding, the *Waste management* process, which plays a crucial role in the P budget, does not possess the same level of information as the production and import-export sectors. The estimation of the P flows in the environment had to rely largely on modeling, despite fairly abundant data availability, owing to the high complexity of the processes involved.

In addition to the partial lack of data, a multiyear MFA faces another problem, namely, the consistency of sources over time, which can further reduce the data availability in comparison to a static 1-year budget. For the current study, an example is the trade of mineral fertilizers for which FAOSTAT had to be discarded as a possible source because its accounting methodology was modified in 2002, giving rise to two incompatible data sets.

The data corresponding to P concentration are largely available and primarily obtained from peer-reviewed literature and scientific reports, although this information was often derived from studies carried out in different countries and distinct years. To a lesser extent than the data regarding the goods, the P concentration also show a decreasing level of information from production and consumption to waste management. Nonetheless, throughout the entire system, this information is usually more detailed than the existing categories of the material flows would require.

### Uncertainty Characterization

The uncertainty of material flow data was characterized using an approach developed by Laner and colleagues (2015), based on the work of Weidema and Wesnæs (1996) and Hedbrant and Sörme (2001). It is a step-wise procedure that starts with the assessment of how representative the data at hand are with respect to the actual values of interest. Similarly to the pedigree matrix proposed by Weidema and Wesnæs (1996), this assessment relies on a set of quality indicators and on the definition of criteria to select evaluation scores. The indicator *Reliability* refers to the documentation of the data and reflects the transparency in regard to the sampling, collection, and verification procedures. *Completeness* describes how complete the available data are in comparison to the actual values of interest. *Temporal correlation* and *Geographical correlation* take into account to which extent the available information diverges from the datum of interest, from a temporal and geographical perspective, respectively. The indicator *Further correlation* reflects deviations other than temporal or geographical, such as parameters of a specific type of technology or process. With respect to the original set of indicators suggested by Laner and colleagues (2015), *Composition* was added in this work to describe the level of information on the composition of the goods, which is of crucial importance for the correct selection of the P concentration, especially in the case of heterogeneous or aggregated flows. For each indicator, four evaluation scores are available, where 1 represents the best level and 4 the worst. The indicators and the criteria defined to assign the evaluation scores are reported in table 1. Whereas the indicators *Reliability* and *Completeness* play a decisive role for any type of data, the relative importance of the other indicators changes from case to case; therefore, their contribution to the final uncertainty also considers the level of sensitivity of the data, defined as the degree of influence of the deviation (e.g., temporal or geographical) from the existing datum to the value of interest. Three levels of sensitivity are defined: *Highly sensitive*, *Sensitive*, and *Not sensitive* for which a deviation of the datum is highly relevant, relevant, or not relevant, respectively. The evaluation scores of the data quality indicators are directly translated into coefficients of variation (CVs) through continuous exponential-type functions, which also allow the use of intermediate scores. As shown by Laner and colleagues (2015), the selection of the functions is critical, in that it largely determines the uncertainty ranges. The reason for choosing exponential-type curves is the fact that these have been the most used functions in the past, among others by Hedbrant and Sörme (2001), on which this approach builds. Another important decision lies in the selection of the parameters that describe the functions. These need to be determined by the modelers, in order to ensure their plausibility according to the specificities of each case study. In this work, the mathematical functions illustrated in the supporting information on the Web (S3) were used, which produce the CVs presented in table 2. These values are well in line with the uncertainty levels applied in other P MFA studies, included within the ranges 1.5% to 50% (Klinglmair et al. Forthcoming), 7% to 51% (Van Dijk et al. 2015), 10% to 100% (Egle et al. 2014), 5% to 100% (Ott and Rechberger 2012), 5% to 50% (Neset et al. 2008), and 0% to 50% (Antikainen et al. 2005). Given that each of the indicators represents an independent aspect of the overall data uncertainty and under the assumption of normality, the coefficient of variation CV_a_ that represents the total uncertainty of the input data is calculated through equation 1, where CV_r_, CV_c_, CV_co_, CV_t_, CV_g_, and CV_f_ represent the coefficients of variation of the indicators *Reliability*, *Completeness*, *Composition*, *Temporal correlation*, *Geographical correlation*, and *Further correlation*, respectively.

1 $$ {CV}_{A,i}=\kern0.33em \sqrt{CV_{r,i}^2+{CV}_{c,i}^2+{CV}_{co,i}^2+{CV}_{t,i}^2+{CV}_{g,i}^2+{CV}_{f,i}^2} $$

**Table 1:** Indicators and criteria applied to assess the data quality

		Score		
Indicator	1	2	3	4
Reliability	Methodology of data generation is well documented and consistent (e.g., Standard documentation—Meta information of National Statistics; laboratory analytical methods).	Methodology of data generation is described, but not fully transparent.	Methodology is not described, but principle of data generation is clear.	Methodology of data generation is unknown (data presented without any metainformation).
Completeness	Complete acquisition of data (no extrapolation; for aggregated flows, data available for all goods).	Partially fragmented data (minor need for extrapolation; for aggregated flows, data available for majority of goods).	Fragmented data (considerable need for extrapolation; for aggregated flows, data available for minority of goods).	Highly fragmented data (major need for extrapolation; for aggregated flows, data available for less than one third of the goods).
Composition	Value is expressed in detailed categories (adequate to select correct P concentration for each category) or no categories exist (single/uniform P concentration).	Value is expressed in large categories.	Value is only partially expressed in categories.	No information on the composition is available (no basis to select appropriate P concentration).
Temporal correlation	Value relates to the correct year.	Deviation of 1 to 5 years.	Deviation of 6 to 10 years.	Deviation of more than 10 years.
Geographical correlation	Value relates to the studied region.	Value relates to comparable region/economy/society.	Value relates to less-comparable region/economy/society.	Socioeconomically different region.
Further correlation	Value relates to the same product, the same technology, etc.	Value relates to similar technology, product, etc.	Values deviates from technology/product/…of interest, but still acceptable.	Value deviates strongly from technology…of interest; correlation unknown.
Expert judgment	Formal statement from qualified expert.	Robustly based estimation.	Weakly based estimation.	Speculation or crude assumption.

Note. P = phosphorus.

**Table 2:** Coefficients of variation (%) for quality indicators, according to score and sensitivity level (where it applies)

Score	Sensitivity	1 (%)	2 (%)	3 (%)	4 (%)
Reliability	—	4	10	22	50
Completeness	—	0	10	22	50
Composition					
Temporal correlation	Highly sensitive	0	10	22	50
Geographical correlation	Sensitive	0	5	11	22
Further correlation	Not sensitive	0	2	4	8
Expert judgment	—	10	20	40	80

Note. “Composition,” “Temporal correlation,” “Geographical correlation,” and “Further correlation” are four independent indicators. They all have the same set of coefficients of variation that are determined by the three sensitivity levels (in the second column). That is why the coefficients of variation are aligned to the rows of sensitivity levels and not to the rows of the four indicators.

If no data are available and the input values are determined through expert judgments, the uncertainty is directly assessed through the indicator *Expert judgment*.

This method is partially exposed to subjectivity in two aspects, namely, in the assignment of evaluation scores for the quality indicators and in the choice of the functions that translate the scores into CVs. With the exception of *Temporal correlation*, for which scores are defined through a precise number of years, for the other indicators the choice of scores 1 (best) and 4 (worst) are straightforward, whereas the scores in between are not univocal and are meant to allow the modelers to make use of their own experience and express also subtle differences. With respect to the mathematical functions, the choice is not unique and, in this type of studies, it is very hard or typically impossible to validate them owing to lack of empirical data. Therefore, any comparison of the uncertainties here calculated with uncertainties estimated in other studies shall be carried out with prudence. This notwithstanding, the approach has the advantage of providing the authors with a scheme that, thanks to the use of continuous functions, ensures consistency of uncertainty estimates within the model. Besides the general importance that internal consistency plays in any MFA study owing to its potential effects on the data reconciliation process and on the identification of the most critical data, it is of particular relevance in this work, where the flows, their temporal change, and their uncertainties are subject to a systematic assessment and comparison. In addition, the structured uncertainty characterization provides the readers with a documentation of the rationales and the calculations underlying the uncertainties of the MFA results, which makes it reproducible.

A more detailed description of the method and of its positioning in the context of other existing approaches is available in Laner and colleagues (2015).

### Analysis of the Degree of Change of the Budget

**Figure 2:**
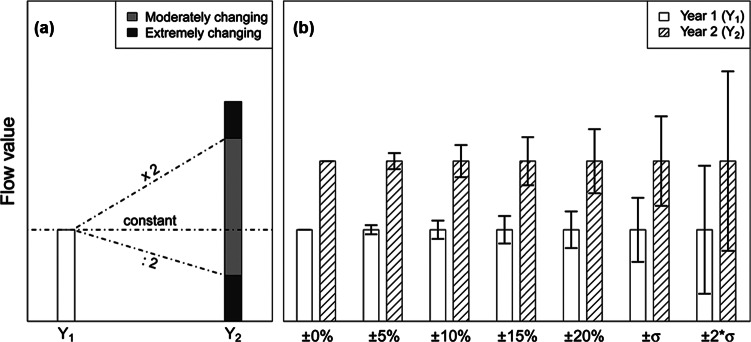
Illustration of the analysis of change in the flows over time: (a) categories of the degree of temporal change and (b) applied tolerance levels to explore impact of different uncertainty levels on capability of detecting the changes. In this example, the indicated change would be rated as moderate for the first five pairs of columns and as constant for the last two (overlapping tolerance levels).

To assess and quantify if and to what extent the system has changed in the 22 years examined in the study, the degree of temporal change for the 122 flows and the eight stock change rates (which can be handled as flows for this purpose) was analyzed and categorized. For each year and each flow, the percentage change was calculated with respect to 1990, the first year of the time series, which was taken as a reference baseline. The presence of null values provokes disproportionate results because the year in which a flow ceases to exist produces a change of –100%, whereas the calculated change when a new flow appears is excessively high. Therefore, in the latter case, an artificial change of 100% was introduced to equally consider the appearance and disappearance of flows. Based on the outcomes, the flows and stock rates were divided into three categories: constant (if no change has occurred); moderately changing; and extremely changing (according to the degree of temporal change). To equally treat increases and decreases, the threshold between the two latter categories was set at the factor 2 that corresponds to a positive change of 100% and to a negative change of –50% (figure 2a).

The following step of this analysis (figure 2b) was designed to investigate whether and to which extent the consideration of the uncertainty would affect the ability of actually detecting the temporal changes. For this purpose, different tolerance levels were defined: ±5%, ±10%, ±15%, and ±20%, these being the ranges within which the temporal changes were ignored (i.e., flows were considered to remain constant). Further, two additional tolerance levels were applied to take into account the specific uncertainty of each flow: ±1 standard deviation and ±2 standard deviations. In this case, the variation of a flow was considered as an actual change only if the respective 68.3% and 95.5% data intervals of the normal distributions characterizing each flow did not overlap.

To evaluate whether the flows have changed gradually or suddenly, the same analysis was carried out by calculating the change of each year with respect to the previous year.

The stocks were excluded from the analysis, partially because their total value is much higher than the contribution of flows of single or even multiple years and partially because of their large initial uncertainty.

### Analysis of the Reconciliation Process

The model was balanced using the software STAN (Cencic and Rechberger 2008). The assumption underlying the algorithm used by STAN is that the parameters are normally distributed independent random variables and that uncertain values are expressed using the mean and the standard deviation. Based on this, the software applies Gaussian error propagation and data reconciliation to alter the mean values and calculate the final uncertainty. Because the standard deviation is used as a weighting factor during the reconciliation process, values with higher uncertainty are more heavily reconciled than values with lower uncertainty. The mathematical details of the calculation can be found in Fellner and colleagues (2011).

The effect of the reconciliation process on the time series was analyzed to assess whether the flows were randomly altered or if the input value was regularly increased or decreased, thus suggesting the presence of a systematic error. If the difference between the input and reconciled values was less than 5%, it was also considered random and therefore not significant for this analysis. Further, the reconciliation of the whole system was also calculated to assess whether there are differences in this respect between the years under study. It was assessed by summing up the percentage variation of the input values of all flows for each year, subsequently normalized with respect to the reconciliation in 1990 to compare the different years. This comparison should also take into account the degree of overdetermination of the model, that is to say, the difference between number of equations and number of unknown variables, but in this case it was not necessary, because the model has the same degree of overdetermination for each year.

## Results and Discussion

### Quality of the Data Set

**Figure 3:**
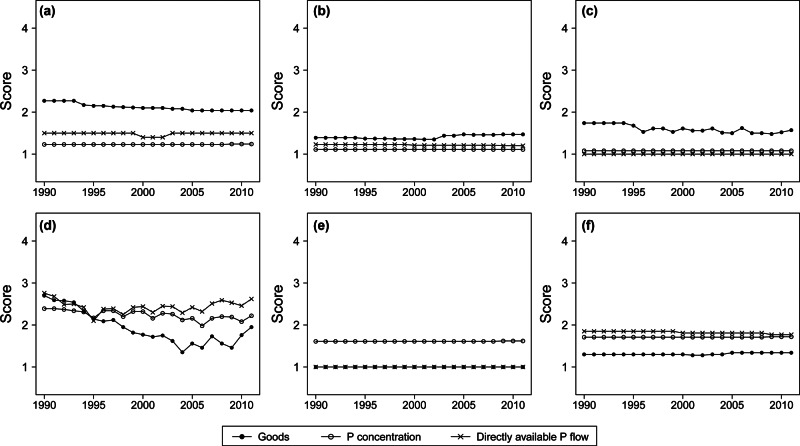
Average scores of the six quality indicators calculated for the goods (mass per time), P concentration, and directly available P flows (P mass per time): (a) reliability; (b) completeness; (c) composition; (d) temporal correlation; (e) geographical correlation; and (f) further correlation. P = phosphorus.

The approach applied in this work for the characterization of data quality is based on a set of indicators (table 1) and on the selection of evaluation scores (1 to 4) for each of them. With the aim of assessing the overall quality of the system, the arithmetical mean of the scores assigned to all flows for each indicator and year was calculated and the outcomes are presented in figure 3. Of the 106 flows for which input values could be quantified, more than 80% were calculated by multiplying goods with their P concentration, whereas for the remainder, the P flows were directly available. Therefore, this evaluation was carried out separately for these types of data.

The *Reliability* (figure 3a), an expression of the level of documentation of the data, was notably good for the P concentration and for the directly available P flows in the past owing to a long tradition of measurements of the P content in several goods. The reliability for the goods was, and still is, poorer but has improved over time, especially in the production and trade statistics, owing to adopted international guidelines and procedures. The lowest level of documentation and transparency was detected for the *Consumption* and *Waste management* processes.

As depicted in figure 3b, incompleteness of the data is not an issue for this study, although a slight quality decrease began near the year 2003 together with the increasing role of the *Bioenergy* process, for which important information is still incomplete.

With respect to the *Composition* indicator, which evaluates the level of detail and categorization of the data, the quality is quite high and has slightly improved over time, although with certain unexpected oscillations on the goods level.

As far as concerns the *Temporal correlation* (figure 3d), for directly available P flows and for P concentration, the average deviation from the datum to the year of interest oscillates around 6 years, whereas for the goods, it has considerably decreased from 10 to less than 5 years. In this respect, the increase after 2009 could be misleading and should not be interpreted as a general decrease of the frequency, because at the time of this study, certain important reports and statistics had not yet been published for the years 2010 and 2011.

The geographical deviation (figure 3e) from the study area affects only the P concentration because the information is often obtained from international literature, although generally from countries similar to Austria.

The indicator *Further correlation* describes all other types of deviations from the actual datum of interest, for example, market year versus calendar year or live weight versus carcass weight. As shown in figure 3f, such deviations are present in the study, although they do not represent a significant problem.

From this evaluation, it can be deduced that the data available for the Austrian national P budget are of generally high quality (average low scores), although it is important to remember that this analysis uses average scores and that data problems for certain specific flows still exist. Moreover, a fraction (however small) of the information required for the calculation of the flows is not available and must be estimated; its corresponding uncertainty was directly quantified through the indicator *Expert judgment* and is therefore not represented in figure 3, which illustrates the quality of the actually available data.

The relative uncertainties estimated for all the flows and time series are available as Supporting Information on the Web (S3).

### Degree of Temporal Change of the Budget

**Figure 4:**
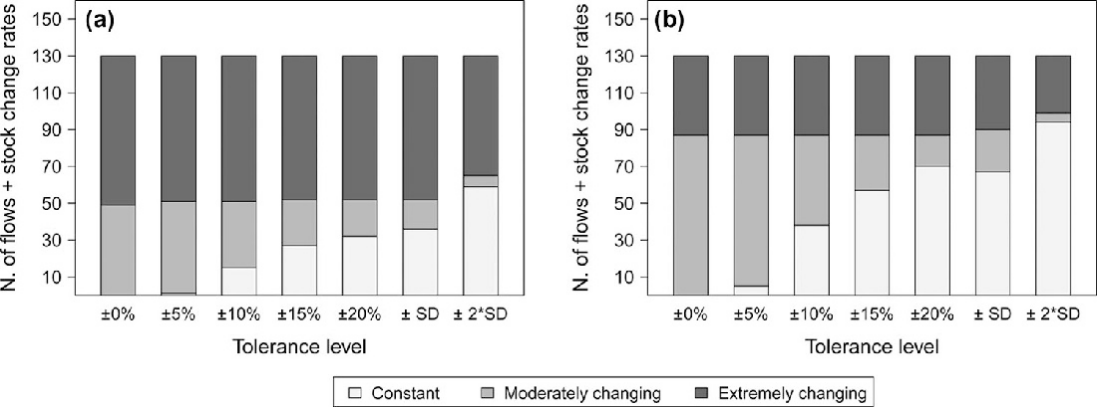
Degree of temporal change of 122 flows and eight stock change rates: (a) categorization according to the change with respect to the reference year 1990 and (b) categorization according to annual change. Results are shown for different tolerance levels (uncertainty thresholds used to determine whether or not temporal changes can actually be detected). The y-axis indicates the number of flows and stock change rates in each category.

Figure 4a shows the outcomes of the analysis of the degree of change of the budget with respect to the reference year 1990. The results are partly sensitive to the applied tolerance levels. If tolerance levels between 0% and ±5% are applied, the analysis indicates that one third of the flows and stock change rates changed moderately, and two thirds were affected by an extreme variation, whereas with ranges from ±10% to ±20%, the fraction of moderately changing flows and stock rates gradually decreases until 15%. The specific standard deviation shows outcomes very similar to the ±20% range, whereas the level of twice the standard deviation decreases both the extreme and moderate fractions to 50% and 5%, respectively. In conclusion, the analysis reveals that half of the flows and stock change rates changed substantially, with certain flows that appeared or disappeared and others that at least doubled or halved their initial value. For the other half, in contrast, it is not possible to ultimately conclude which fraction remained constant or changed moderately from this analysis because of the high uncertainty of the results.

The second component of this analysis instead (figure 4b) explores to what extent the flows and stock change rates changed from a given year to the following one to provide an overview of whether the changes took place gradually or rather abruptly. This analysis predictably and reasonably suggests that a large proportion of the flows were affected by gradual and moderate changes, but between 24% and 33% of the flows (depending on the considered tolerance level) recorded at least one extreme variation, indicating the noteworthy presence of substantial and sudden changes. The outcomes also highlight the difficulty of detecting smaller annual changes when uncertainty ranges are applied. This situation poses a problem in view of P management monitoring.

**Figure 5:**
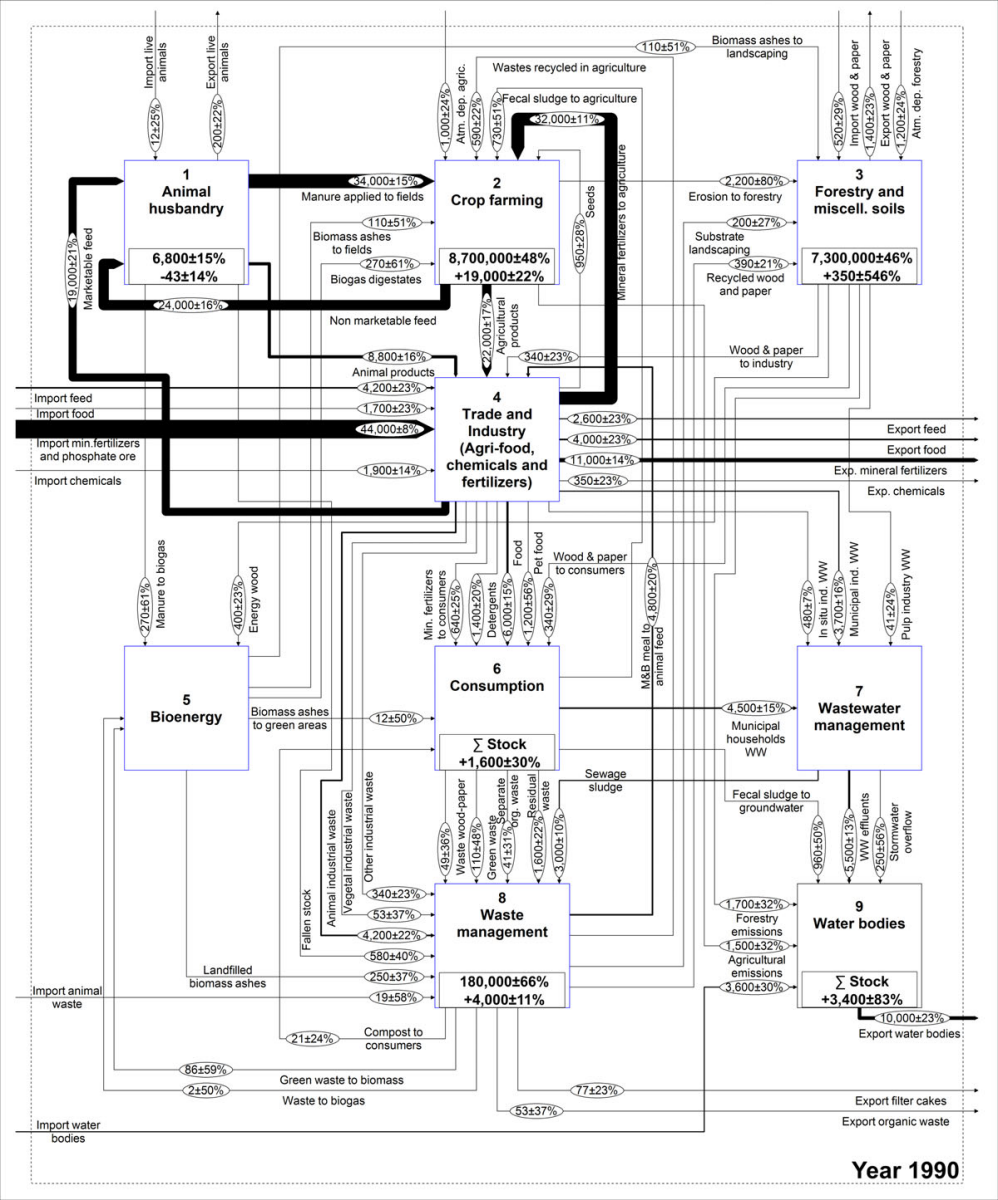
Austrian P budget representing the year 1990. Units for flows and stocks are tonnes/y and tonnes, respectively. P = phosphorus; y = year.

**Figure 6:**
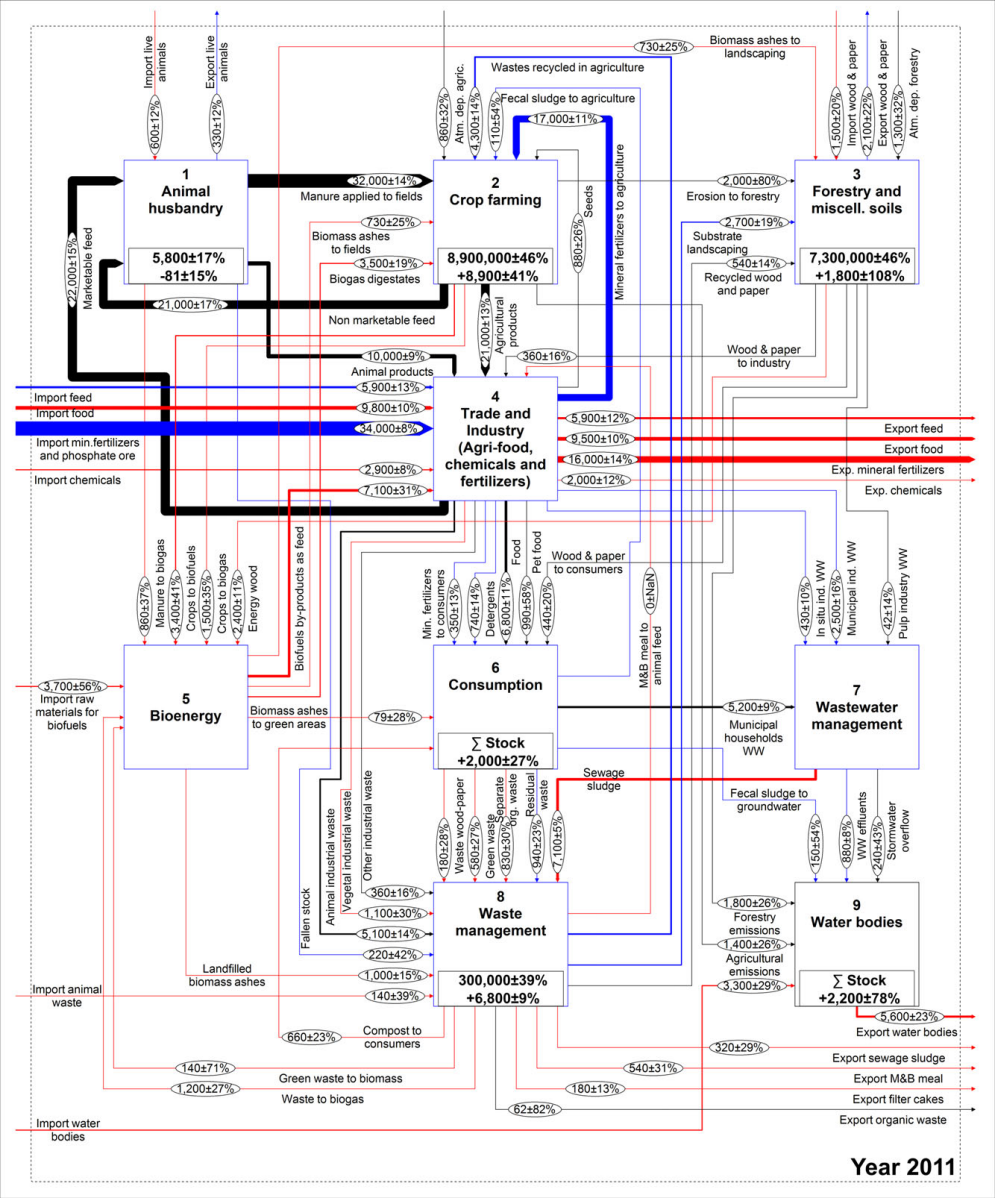
Austrian P budget representing the year 2011. Units for flows and stocks are t/y and t, respectively. The colors represent the change calculated over the entire time series with respect to 1990 (figure 5), taking SD as the tolerance level: (black) constant; (blue) moderately changing; and (red) extremely changing. P = phosphorus; t/y = tonnes per year; t = tonnes; SD = standard deviation.

Figures 5 and 6 show a comparison between the budgets in the year 1990 and in 2011. In the latter, the flows are colored according to the change of the entire time series from 1990 and are based on the standard deviation as the tolerance level. This figure shows that the majority of the extreme changes are connected to industrial trade, sewage sludge, meat and bone meal, and the *Bioenergy* process.

### Long-Term Data Reconciliation

**Figure 7:**
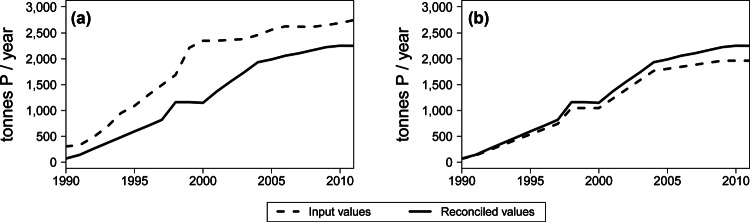
Comparison between input and reconciled values of the *Composting* subprocess: (a) sum of input flows and (b) sum of output flows.

The analysis of the data reconciliation process on the time series reveals that less than 10% of the flows were systematically reconciled, that is to say, they were always altered in the same direction. This result indicates the presence of systematic errors, however small. The *Composting* subprocess, which is a subset of the *Waste management* compartment, offers a clear example. As shown in figure 7, over the entire period under study, the input flows were reduced, and the output flows were increased by the reconciliation process. Two potential sources of error lie in the fact that the data on the composted sewage sludge are often aggregated together with data on sludge handled by other mechanical-biological treatments, and that the P content of the compost products is quite heterogeneous. Owing to the lack of further information, it is not possible to identify the actual cause of the problem, but at least the systematic inconsistency was detected and could be addressed in the future if more accurate data are made available. The analysis of the reconciliation process was carried out not only at the end of the study, but was also included as an additional step within the usual iterative procedure behind MFA, as described by Laner and colleagues (2014). This action has aided in identifying errors, which could be solved by either gathering more detailed data or correcting estimations, thus leading to an improved database for the budget.

With respect to the entire system, the results (available as Supporting Information on the Web [S4]) show that the impact of the reconciliation on the input values has decreased in time owing to a general improvement of the data quality. Notwithstanding this general improvement, the impact of the reconciliation presents fluctuations in the time series, showing that the quality of a static MFA is sensitive to the choice of the year for which it is carried out.

### Selected Time Series Relevant to Sustainable Management

As previously shown by Egle and colleagues (2014) and in line with the trend in Western European countries (Schröder et al. 2011), the use of mineral fertilizers was remarkably reduced over the last 20 years. In addition, in the same period, the Austrian livestock population has constantly decreased; therefore, the accumulation of P in agricultural soils was further reduced. Nonetheless, the surplus accumulated in the past decades was so high that no impact on crop production or emissions to water bodies was visible, which is in agreement with the findings of Senthilkumar and colleagues (2012) for France.

The results show that the average daily intake of P of the Austrian population has remained practically constant with an average value of 1.6 gP/cap*d (grams of phosphorus per capita per day), in agreement with the findings of Welch and colleagues (2009) for Germany and of Elmadfa (2009) for Central and Eastern Europe, which does exceed the values recommended by DGE and colleagues (2003). The actual intake can diverge considerably from food availability, depending on the degree of loss of edible food and nutrients through the consumption process. This study reveals that the available P that is wasted was already approximately 30% in 1990 and increased up to 40% by 2011. What has improved in this respect is the recovered fraction of the waste P from the *Consumption* process, which has increased from approximately 20% in 1990 to circa 60% in 2011, primarily owing to the enhanced separate collection of organic waste. To a lesser degree, the improvement was also impacted by more widespread home composting, which is not an indicator of better management per se given the potential accumulation of unnecessary P in gardens and green areas, as discussed in Egle and colleagues (2014). An important aspect that has emerged from this study with respect to food P is the trend of rapidly increasing import, from 1,700 ± 23% tonnes of phosphorus (tP) in 1990 to 9,800 ± 10% tP in 2011. This amount is relevant in terms of material accounting and environmental and resource management because it poses the question of how to measure, control, or even take into account the mineral P consumption and the pollution taking place in other countries.

Whereas biomass thermal plants have a longer tradition in Austria, the biogas and biofuels sectors (with the exception of few rural and small plants) have recently emerged in the years 2002–2003 and were characterized by notably quick expansion with a consumption of 1,200 ± 32% tP contained in crops in the year 2004, which grew to 8,600 ± 30% tP in 2011. Further, they currently handle approximately 1,000 tP per year (tP/yr) of industrial organic waste and circa 800 tP/yr of manure. Biogas, bioethanol, and biodiesel contain practically no P. Therefore, a component returns to the agricultural fields under the form of biogas digestates, whereas the remainder ends up in biofuels by-products, which are typically used as animal feed. In the United States, where biofuels production is rapidly growing, this new stream of P-rich animal feed is creating concerns because it could undermine efforts toward feed management practices designed to reduce the P load in manure (Simpson et al. 2008). This study estimates that 7,100 ± 31% tP were contained in the stream of these by-products in 2011, which is noteworthy if compared with the total P in animal feed consumption of 42,800 ± 12% tP. Nevertheless, the only datum available for this study was the generation of dried distiller's grains, that is to say, bioethanol by-products, whereas the estimations of the biodiesel by-products and their final use relied on indirect information and assumptions.

**Figure 8:**
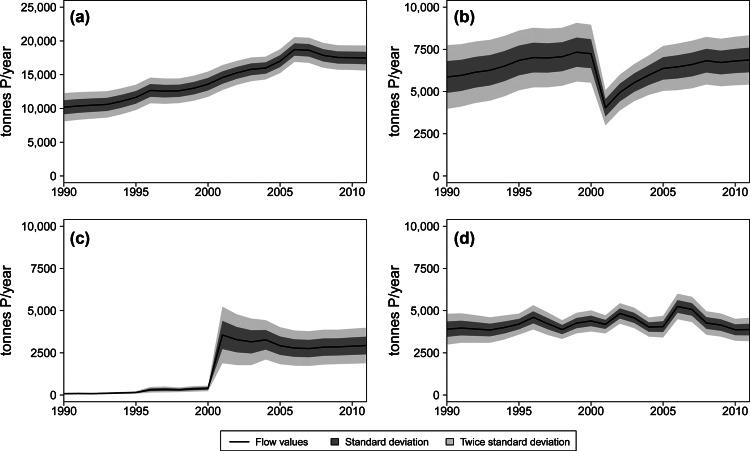
Time series of flows and stock change rates reproduced with their specific uncertainty: (a) total P input in the *Waste management* process; (b) total P recovery within the *Waste management* process; (c) total P used in cement kilns; and (d) total disposal of P in landfills. P = phosphorus.

The total input of P into the *Waste management* process, the time series of which is illustrated in figure 8a, encompasses the entire generation of solid waste from households and similar sources, industry and green areas, fallen stock from *Animal husbandry*, total sewage sludge generated in wastewater treatment plants, disposed ashes generated in biomass thermal plants, and imported waste. Items that are not included are home-composted kitchen and green residues and organic by-products of the food industry directly reused for the production of animal feed, which are both separately accounted as internal flows of the processes *Consumption* and *Industry*. The total amount of P transported in waste flows has constantly and significantly increased from 1990 to 2011. Population growth only partially explains this trend because the waste P expressed per capita was augmented from 1.2 to 1.5 kilograms (kg) P in 1990 to 2.0 to 2.2 kg P in 2011. The main determinant was sewage sludge, which increased from 3,000 ± 11% tP in 1990 to 7,100 ± 6% tP in 2011 owing to population growth, incremented connection rates, and, particularly, highly enhanced P removal in treatment plants. A less crucial, but still important, role was played by organic industrial waste, expressed by the growing food industry as well as disposed biomass ashes, which are indicative of the development of the bioenergy sector.

Figure 8b illustrates the fraction of the input wastes that are recovered, namely, meat and bone meal used as animal feed, materials (sewage sludge, compost, and meat and bone meal) applied on agricultural fields, recycled wood and paper, compost applied in gardens and green areas, wastes valorized in biogas plants, and green residues used in biomass thermal plants. The fraction of waste used in landscaping activities is excluded because it does not necessarily imply a useful recovery of P, as well as the exported fraction for which the final management or disposal information is not always known. In the 1990s, approximately 5,000 tP in meat and bone meal per year were recovered as animal feed, but in 2001, this use was banned, which explains the drastic drop in figure 8b. The total amount of recovered P showed a rapid increase after 2001, primarily owing to the application of processed animal fat and meal on agricultural fields. Nevertheless, the ratio of total recovery with respect to the total input of waste has decreased from 55% to 60% in 1990 to approximately 40% in 2011, showing that in terms of P, waste generation has grown faster than recycling. A large fraction of the total waste P, approximately 40% to 45% on average, has been lost in cement kilns and landfills, with oscillations ranging between 35% and 55% in different years. In addition to the minor contribution of sewage sludge, the P is mostly lost in cement kilns through the use of meat and bone meal as a secondary fuel; therefore, this flow was almost null until 2001, when it suddenly reached notably high values of approximately 4,000 tP/yr, which was gradually reduced to approximately 3,000 tP/yr in more recent years (figure 8c). The amount of P deposited in landfills has not shown important variations in the period under study and has remained in the range of 4,000 to 6,000 tP/yr, as illustrated in figure 8d. Nevertheless, the material streams transporting it have varied considerably. In 1990, the P was mainly landfilled through direct disposal of sewage sludge, residual waste, and ashes of municipal waste incineration and sludge coincineration, but in 2011, the main flows in order of importance were ashes of municipal waste incineration, sludge coincineration, and thermal biomass plants, followed by sludge and municipal waste stabilized through mechanical-biological processes. The time series in figure 8, which are depicted together with the uncertainty, show the differences in the level of information behind different flows within the *Waste management* process. The total input (figure 8a) presents less uncertainty than the recovery (figure 8b) and the loss in cement kilns (figure 8c), because the report for the generation of waste is more detailed in general than for its management. With respect to the loss in landfills (figure 8d), the uncertainty is significantly lower and decreasing in time because the disposal of organic waste and sewage sludge has been specifically regulated and monitored.

## Conclusions

Even over a relatively short and stable period of 22 years, the national P budget of Austria has undergone significant and partially abrupt changes that have affected different compartments of the system. In the last decade, the bioenergy sector has gained increasing importance and now affects large flows of raw materials, waste, and by-products. This rapid growth was, however, not accompanied by a sound monitoring of the associated flows, which shall be implemented because such flows could negatively affect the P balance in agriculture, especially in animal husbandry activities. With respect to P recovery, although in household wastes it has considerably increased from 20% in 1990 to 60% in 2011, in total wastes it has declined from 55% to 60% to approximately 40%. In this context, the sudden and large loss of P contained in the meat and bone meal and diverted since 2001 to cement kilns, as a consequence of the Bovine Spongiform Encephalopathy crisis, represents a clear example of the impact that apparently unrelated modifications can exert on the P budget.

If the uncertainty of the results is considered in the analysis of temporal changes, extreme changes over the entire period are still clearly visible, but for half of the flows it is difficult to determine whether they remained constant or if moderate changes took place. The study also shows that the ability to detect annual variations is very sensitive to the uncertainty of the data set and this is highly relevant for the monitoring of the performance of future measures or policies.

From a methodological point of view, this study has improved the model, in that it has aided the detection of systematic problems and inconsistencies in the data sets, which could have not been revealed by a 1-year static study.

This exercise has demonstrated that it is feasible to carry out a time series of a detailed national budget, it has identified temporal developments with relevant implications for environmental, resources, and waste management, and it has shown manifold methodological benefits and added values.

Given all the aforesaid, this study suggests the need and usefulness for national authorities to set up similar resource accounting schemes, which could provide different services. First, they could identify and detect negative trends of flows, stocks, efficiencies, and recovery rates. Second, they could monitor the effectiveness of regulations and directives. Last, they could aid in optimizing the national data collection by identifying the specific contribution of different areas to the uncertainty of the system.

## Supplementary Information

**Supporting Information S1**: This supporting information provides visualizations of the MFA model subsystems.

Supporting info item

**Supporting Information S2**: This supporting information provides descriptions of flows, stocks, transfer coefficients and equations for their calculation, and data sources.

**Supporting Information S3**: This supporting information characterizes data uncertainty.

**Supporting Information S4**: This supporting information displays the degree of impact that reconciliation has had on the whole system.

## References

[CR1] Antikainen, R., R. Leemola, J. I. Nousiainen, L. Sokka, M. Esala, P. Huhtanen, and S. Rekolainen. 2005. Stocks and flows of nitrogen and phosphorus in the Finnish food production and consumption system. *Agriculture, Ecosystems & Environment* 107(2): 287–305.

[CR2] Brunner, P. H. and H. Rechberger. 2004. *Practical handbook of material flow analysis*. Boca Raton, FL, USA: CRC/Lewis.

[CR3] Cencic, O. and H. Rechberger. 2008. Material flow analysis with software STAN. *Journal of Environmental Engineering and Management* 18(1): 3–7.

[CR4] Chowdhury, R. B., G. A. Moore, A. J. Weatherley, and M. Arora. 2014. A review of recent substance flow analyses of phosphorus to identify priority management areas at different geographical scales. *Resources, Conservation and Recycling* 83: 213–228.

[CR6] Condron, L. M., B. M. Spears, P. M. Haygarth, B. L. Turner, and A. E. Richardson. 2013. Role of legacy phosphorus in improving global phosphorus-use efficiency. *Environmental Development* 8: 147–148.

[CR7] Cooper, J., R. Lombardi, D. Boardman, and C. Carliell-Marquet. 2011. The future distribution and production of global phosphate rock reserves. *Resources, Conservation and Recycling* 57: 78–86.

[CR8] Cordell, D., J.-O. Drangert, and S. White. 2009. The story of phosphorus: Global food security and food for thought. *Global Environmental Change* 19(2): 292–305.

[CR9] Cordell, D., T.-S. S. Neset, and T. Prior. 2012. The phosphorus mass balance: identifying “hotspots” in the food system as a roadmap to phosphorus security. *Current Opinion in Biotechnology* 23(6): 839–845. 22503084 10.1016/j.copbio.2012.03.010

[CR10] Cordell, D. and S. White. 2011. Peak phosphorus: Clarifying the key issues of a vigorous debate about long-term phosphorus security. *Sustainability* 3(10): 2027–2049.

[CR11] DGE, ÖGE, SGE, and SVE. 2003. *Referenzwerte für die Nährstoffzufuhr* [Reference values for nutrient intake]. 1. Auflage, 5., korrigierter Nachdruck. Bonn, Germany: UMSCHAU (German).

[CR12] Edixhoven, J. D., J. Gupta, and H. H. G. Savenije. 2013. Recent revisions of phosphate rock reserves and resources: Reassuring or misleading? An in-depth literature review of global estimates of phosphate rock reserves and resources. *Earth System Dynamics* 4(2): 1005–1034.

[CR13] Egle, L., O. Zoboli, S. Thaler, H. Rechberger, and M. Zessner. 2014. The Austrian P budget as a basis for resource optimization. *Resources, Conservation and Recycling* 83: 152–162.

[CR14] Elmadfa, I. 2009. *European nutrition and health report 2009*. Vienna: Forum of Nutrition. 10.1159/000242365 20081326

[CR15] European Commission. 2014. The European Critical Raw Materials review. MEMO/14/377. May 26. http://europa.eu/rapid/press-release_IP-14-599_en.htm. Accessed 17 July 2014.

[CR16] Fellner, J., P. Aschenbrenner, O. Cencic, and H. Rechberger. 2011. Determination of the biogenic and fossil organic matter content of refuse-derived fuels based on elementary analyses. *Fuel* 90(11): 3164–3171.

[CR17] Fischer Kowalski, M., F. Krausmann, S. Giljum, S. Lutter, A. Mayer, S. Bringezu, Y. Moriguchi, H. Schütz, H. Schandl, and H. Weisz. 2011. Methodology and indicators of economy-wide material flow accounting. *Journal of Industrial Ecology* 15(6): 855–876.

[CR18] Hedbrant, J. and L. Sörme. 2001. Data vagueness and uncertainties in urban heavy-metal data collection. *Water, Air and Soil Pollution: Focus* 1(3–4): 43–53.

[CR19] Kauwenbergh, S. J. Van. 2010. *World phosphate rock reserves and resources*. Muscle Shoals, AL, USA: IFDC.

[CR20] Klinglmair, M., C. Lemming, L. S. Jensen, H. Rechberger, T. F. Astrup, and C. Scheutz. Forthcoming. Phosphorus in Denmark: National and regional anthropogenic flows. *Resources, Conservation and Recycling*.

[CR21] Koppelaar, R. H. E. M. and H. P. Weikard. 2013. Assessing phosphate rock depletion and phosphorus recycling options. *Global Environmental Change* 23(6): 1454–1466.

[CR22] Lamprecht, H., D. J. Lang, C. R. Binder, and R. W. Scholz. 2011. The trade-off between phosphorus recycling and health protection during the BSE crisis in Switzerland. A “disposal dilemma.” *GAIA—Ecological Perspectives for Science and Society* 20(2): 112–121.

[CR23] Laner, D., J. Feketitsch, H. Rechberger, and J. Fellner. 2015. A novel approach to characterize data uncertainty in MFA and its application to plastic flows in Austria. *Journal of Industrial Ecology*. DOI: 10.1111/jiec.12326.

[CR24] Laner, D., H. Rechberger, and T. Astrup. 2014. Systematic evaluation of uncertainty in material flow analysis. *Journal of Industrial Ecology* 18(6): 859–870. 10.1111/jiec.12381 PMC5217078 28133432

[CR25] Li, G.-L., X. Bai, S. Yu, H. Zhang, and Y.-G. Zhu. 2012. Urban phosphorus metabolism through food consumption. *Journal of Industrial Ecology* 16(4): 588–599.

[CR26] Ma, D., S. Hu, D. Chen, and Y. Li. 2012. Substance flow analysis as a tool for the elucidation of anthropogenic phosphorus metabolism in China. *Journal of Cleaner Production* 29–30: 188–198.

[CR27] Mohr, S. E. 2013. Projections of future phosphorus production. *Philica*. www.philica.com/display_article.php?article_id=380. Accessed June 2014.

[CR28] Müller, E., L. M. Hilty, R. Widmer, M. Schluep, and M. Faulstich. 2014. Modeling metal stocks and flows: A review of dynamic material flow analysis methods. *Environmental Science & Technology* 48(4): 2102–2113. 24494583 10.1021/es403506a

[CR29] Neset, T.-S. S., H.-P. Bader, R. Scheidegger, and U. Lohm. 2008. The flow of phosphorus in food production and consumption—Linköping, Sweden, 1870–2000. *Science of the Total Environment* 396(2–3): 111–120. 18377956 10.1016/j.scitotenv.2008.02.010

[CR30] Ott, C. and H. Rechberger. 2012. The European phosphorus balance. *Resources, Conservation, Recycling* 60: 159–172.

[CR31] Rechberger, H., O. Cencic, and R. Frühwirth. 2014. Uncertainty in material flow analysis. *Journal of Industrial Ecology* 18(2): 159–160.

[CR32] Schipper, W. 2014. Phosphorus: Too big to fail. *European Journal of Inorganic Chemistry* 2014(10): 1567–1571.

[CR33] Scholz, R. W. and F.-W. Wellmer. 2013. Approaching a dynamic view on the availability of mineral resources: What we may learn from the case of phosphorus? *Global Environmental Change* 23(1): 11–27.

[CR34] Schröder, J. J., A. L. Smit, D. Cordell, and A. Rosemarin. 2011. Improved phosphorus use efficiency in agriculture: A key requirement for its sustainable use. *Chemosphere* 84(6): 822–831. 21349568 10.1016/j.chemosphere.2011.01.065

[CR35] Senthilkumar, K., T. Nesme, A. Mollier, and S. Pellerin. 2012. Conceptual design and quantification of phosphorus flows and balances at the country scale: The case of France. *Global Biogeochemical Cycles* 26(2): GB2008.

[CR36] Simpson, T. W., A. N. Sharpley, R. W. Howarth, H. W. Paerl, and K. R. Mankin. 2008. The new gold rush: Fueling ethanol production while protecting water quality. *Journal of Environment Quality* 37(2): 318. 10.2134/jeq2007.0599 18268293

[CR37] Sverdrup, H. U. and K. V. Ragnarsdottir. 2011. Challenging the planetary boundaries II: Assessing the sustainable global population and phosphate supply, using a systems dynamics assessment model. Ninth International Symposium on the Geochemistry of the Earth's Surface (GES-9. *Applied Geochemistry* 26(S1): S307–S310.

[CR38] Ulrich, A. E. and E. Schnug. 2013. The modern phosphorus sustainability movement: A profiling experiment. *Sustainability* 5(11): 4523–4545.

[CR39] Ulrich, A. E., M. Stauffacher, P. Krütli, E. Schnug, and E. Frossard. 2013a. Tackling the phosphorus challenge: Time for reflection on three key limitations. *Environmental Development* 8: 137–144.

[CR40] Ulrich, A. E., M. Stauffacher, P. Krütli, E. Schnug, and E. Frossard. 2013b. Response to the comments on “Tackling the phosphorus challenge: Time for reflection on three key limitations.” *Environmental Development* 8: 149–151.

[CR41] Vaccari, D. A. and N. Strigul. 2011. Extrapolating phosphorus production to estimate resource reserves. *Chemosphere* 84(6): 792–797. 21440285 10.1016/j.chemosphere.2011.01.052

[CR42] Van der Voet, E. 2002. Substance flow analysis methodology. In *A handbook of industrial ecology*, edited by R. U. Ayres and L. W. Ayres. Cheltenham, UK: Edward Elgar.

[CR43] Van Dijk, K., C. Lesschen, J. Peter, and O. Oenema. 2015. Phosphorus flows and balances of the European Union Member States. *Science of the Total Environment* DOI:10.1016/j.scitotenv.2015.08.048. 10.1016/j.scitotenv.2015.08.048 26421756

[CR44] Vuuren, D. P. Van, A. F. Bouwman, and A. H. W. Beusen. 2010. Phosphorus demand for the 1970–2100 period: A scenario analysis of resource depletion. *Global Environmental Change* 20(3): 428–439.

[CR45] Weidema, B. P. and M. S. Wesnæs. 1996. Data quality management for life cycle inventories—An example of using data quality indicators. *Journal of Cleaner Production* 4(3–4): 167–174.

[CR46] Welch, A. A., H. Fransen, M. Jenab, M. C. Boutron-Ruault, R. Tumino, C. Agnoli, U. Ericson, et al. 2009. Variation in intakes of calcium, phosphorus, magnesium, iron and potassium in 10 countries in the European Prospective Investigation into Cancer and Nutrition study. *European Journal of Clinical Nutrition* 63(S4): S101–S121. 19888269 10.1038/ejcn.2009.77

