# Supplementary material for: Added Values of Time Series in Material Flow Analysis: The Austrian Phosphorus Budget from 1990 to 2011
Source: J Ind Ecol. 2015 Dec 22;20(6):1334–48. doi: 10.1111/jiec.12381 (PMC5217078; doi:10.1111/jiec.12381)
Supplement: Supplementary file 1 — Supporting info item [file 44498_2016_2006008_MOESM1_ESM.pdf]

SUPPORTING INFORMATION FOR:

Zoboli, O., D. Laner, M. Zessner, and H. Rechberger. 2015. Added values of time series in MFA: The Austrian phosphorus budget from 1990 to 2011. *Journal of Industrial Ecology.*

Summary

This supporting information provides visualizations of the MFA model sub-systems.

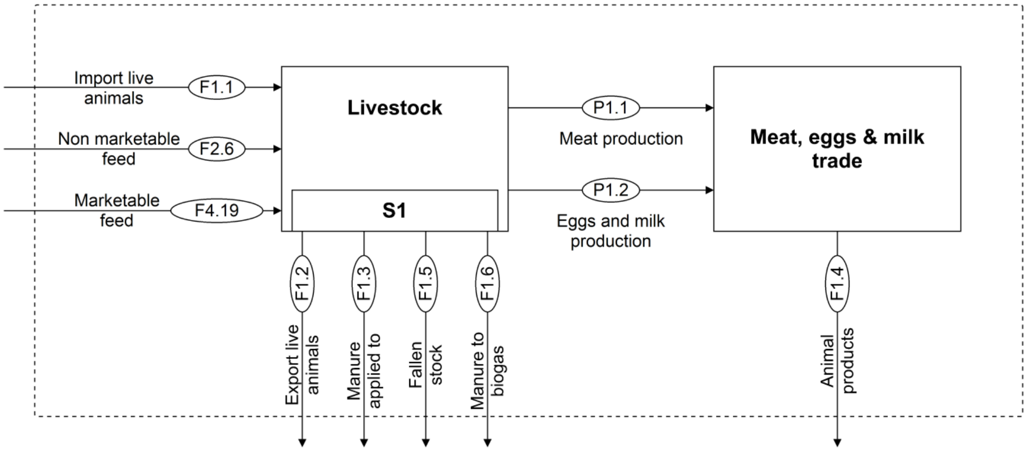

Figure S1-1: MFA model of the *Animal husbandry* subsystem

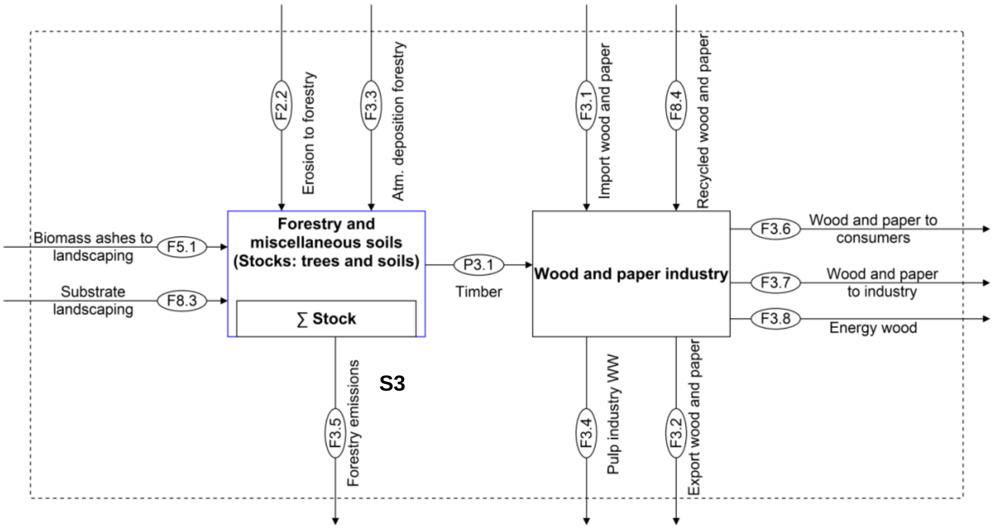

Figure S1-2: MFA model of the *Forestry and miscellaneous soils* subsystem

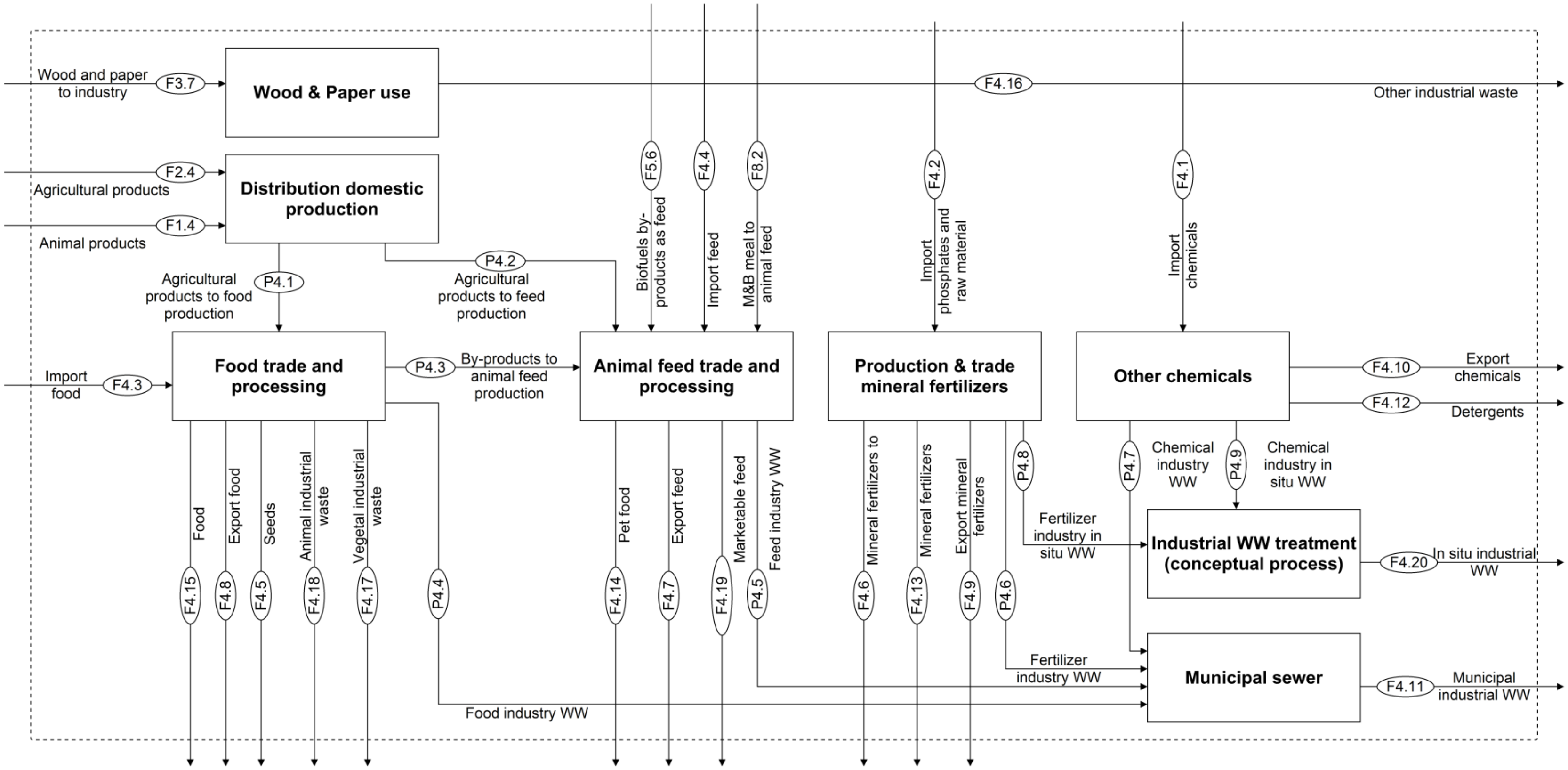

Figure S1-3: MFA model of the *Industry* subsystem

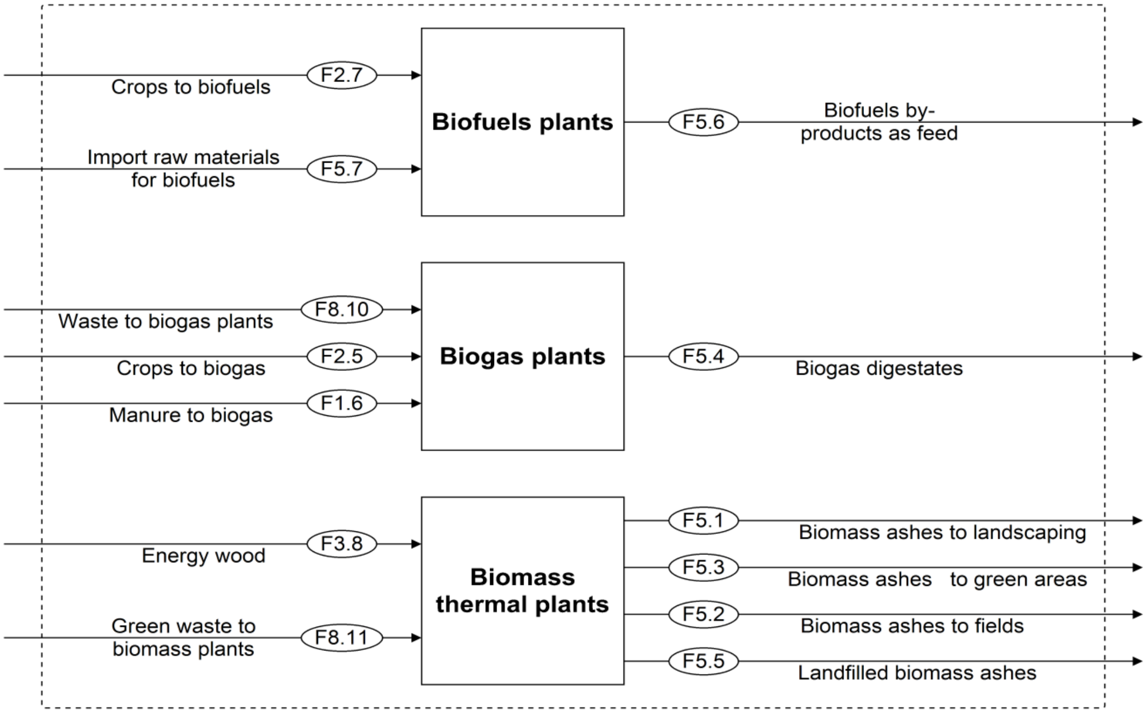

Figure S1-4: MFA model of the *Bioenergy* subsystem

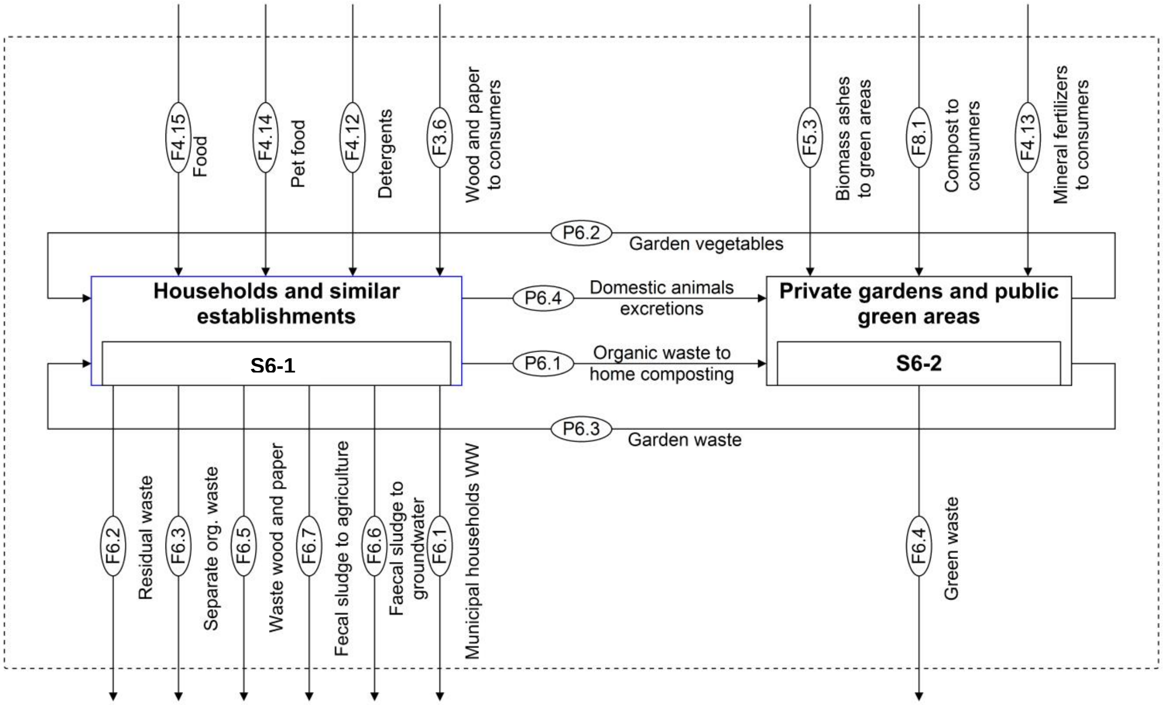

Figure S1-5: MFA model of the *Consumption* subsystem

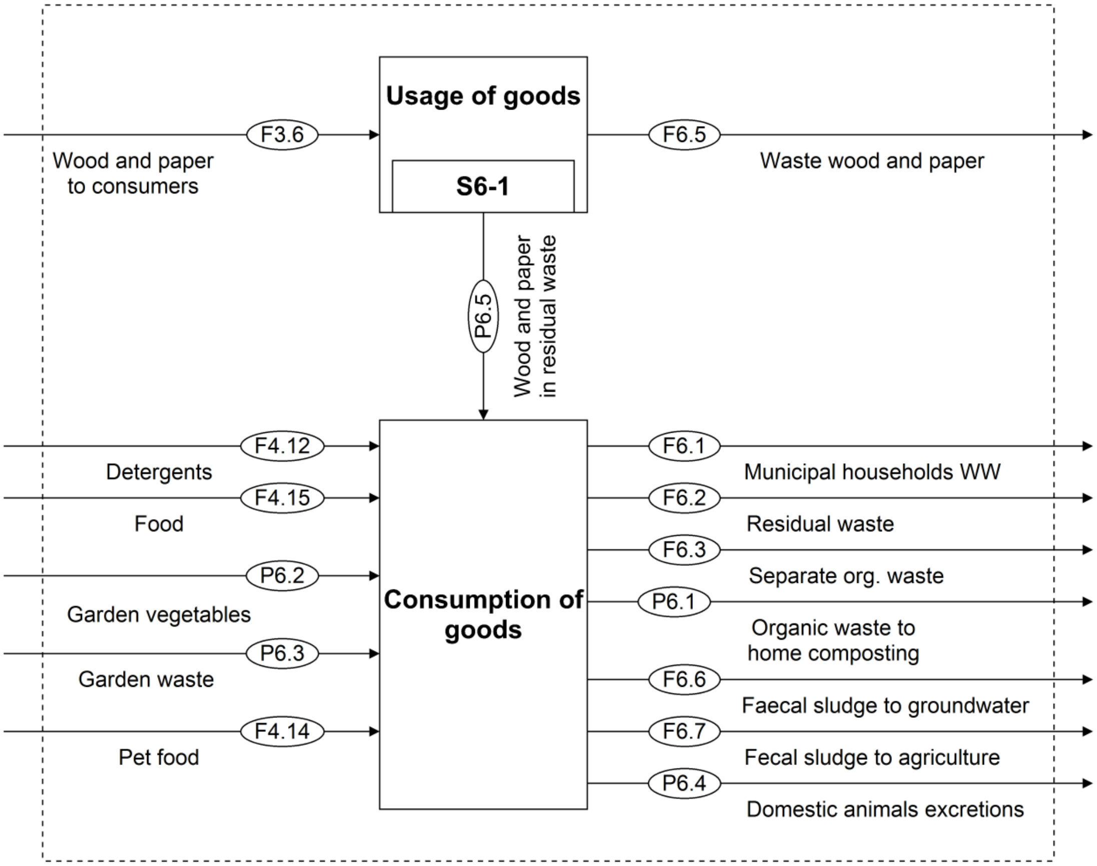

Figure S1-6: MFA model of the *Households and similar establishments* second level subsystem

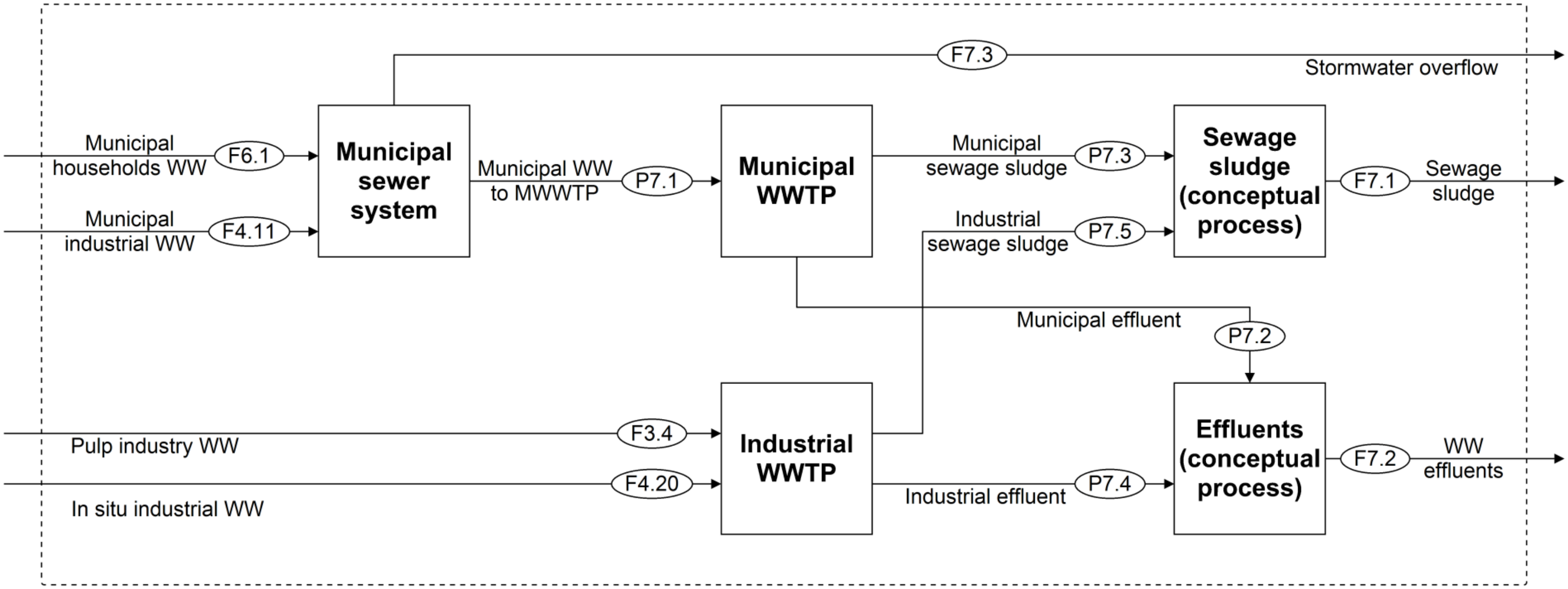

Figure S1-7: MFA model of the *Wastewater management* subsystem

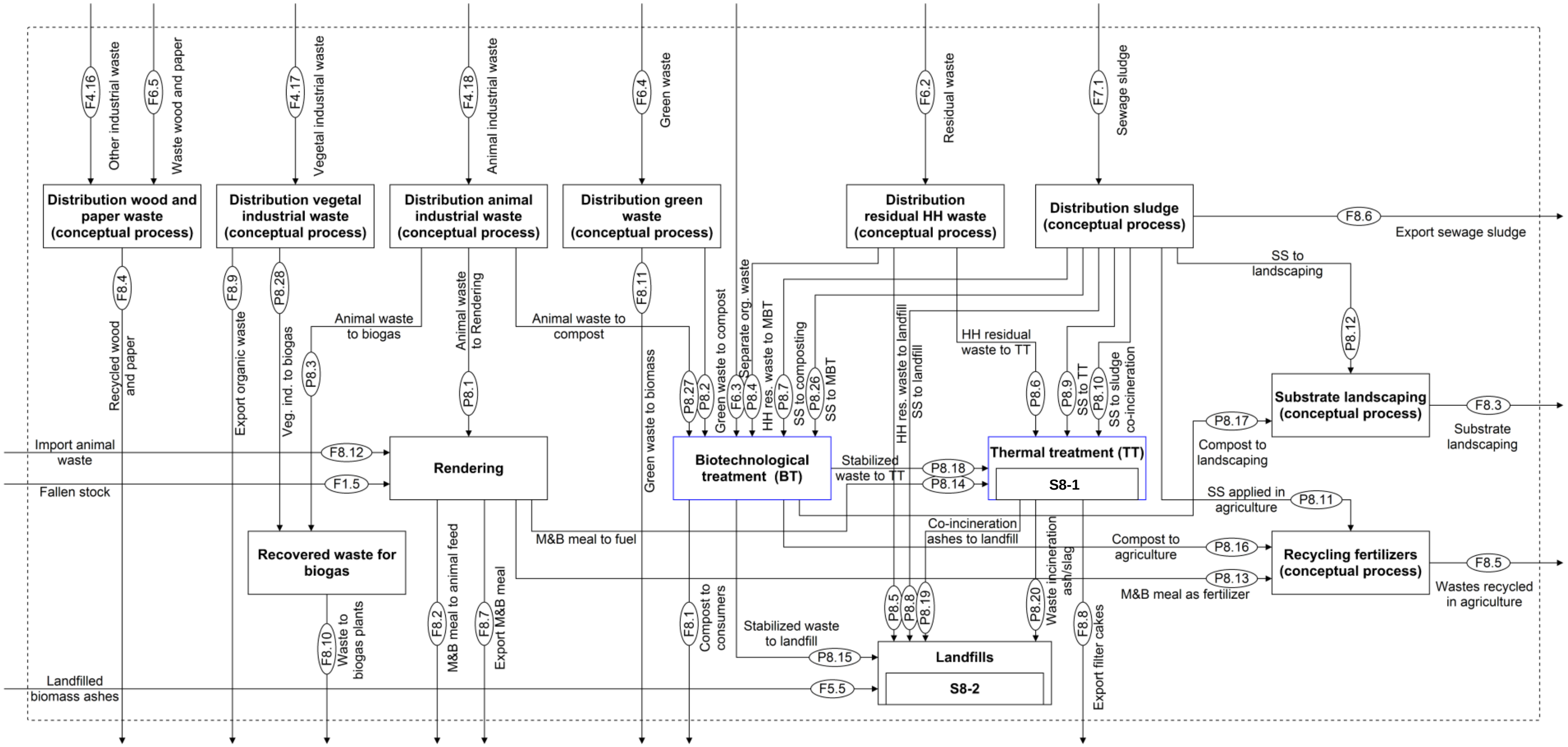

Figure S1-8: MFA model of the W*aste management* subsystem

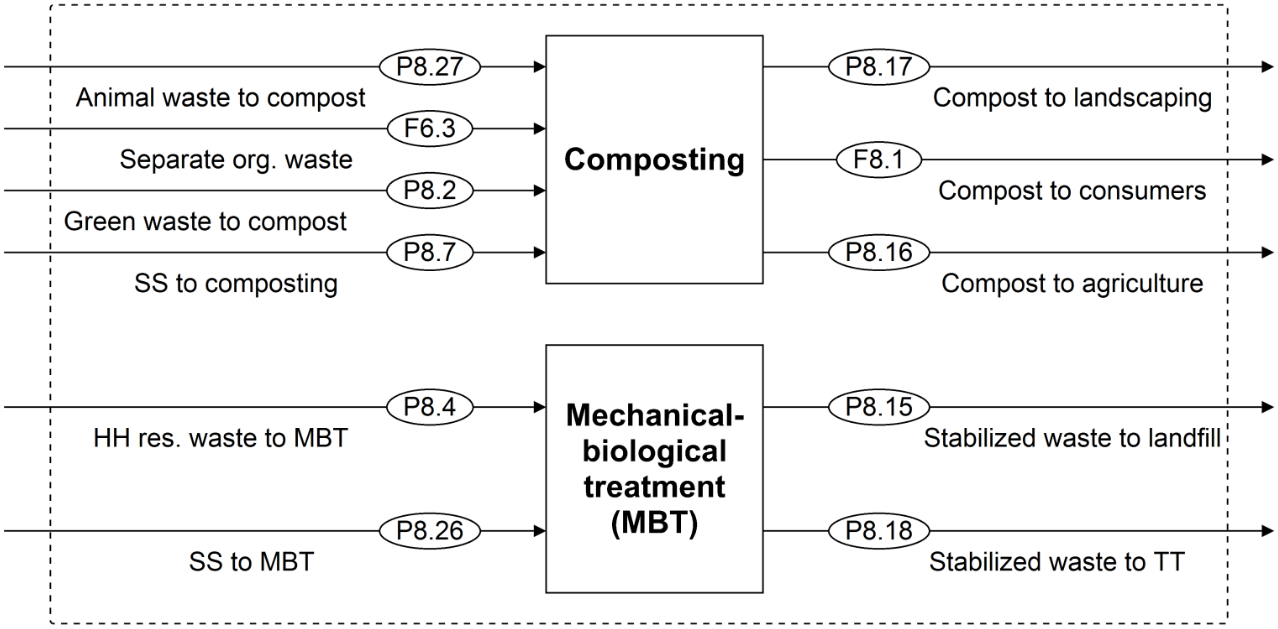

Figure S1-9: MFA model of the *Biotechnological treatment (BT)* second level subsystem

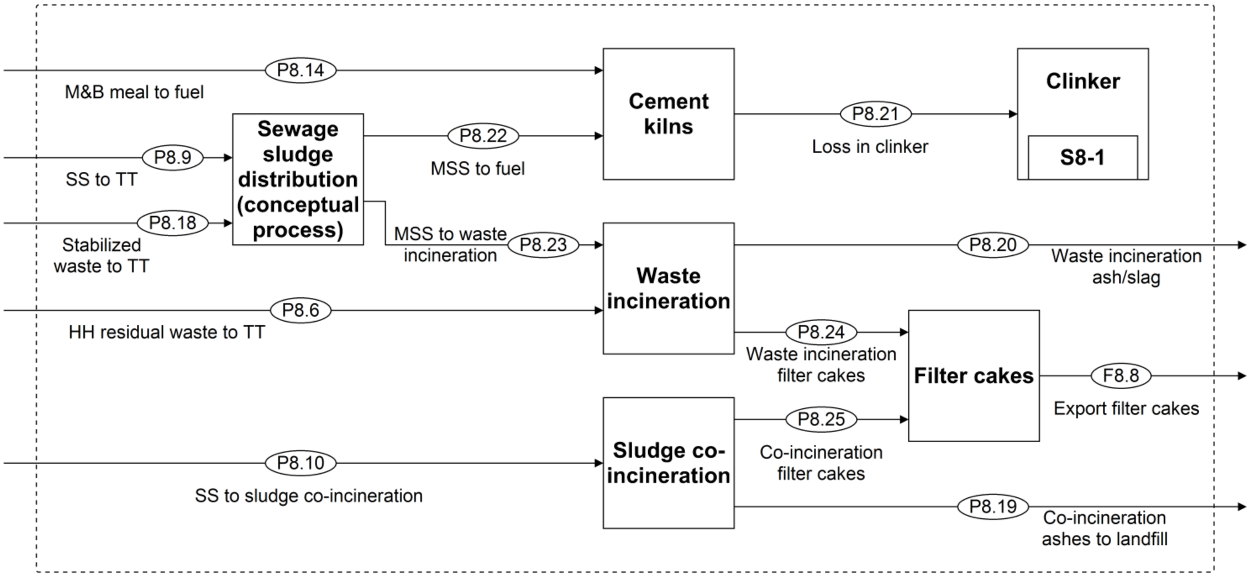

Figure S1-10: MFA model of the *Thermal treatment (TT)* second level subsystem
