# Supplementary material for: Added Values of Time Series in Material Flow Analysis: The Austrian Phosphorus Budget from 1990 to 2011
Source: J Ind Ecol. 2015 Dec 22;20(6):1334–48. doi: 10.1111/jiec.12381 (PMC5217078; doi:10.1111/jiec.12381)
Supplement: Supplementary file 2 — Supporting Information S2: This supporting information provides descriptions of flows, stocks, transfer coefficients and equations for their calculation, and data sources. [file 44498_2016_2006008_MOESM2_ESM.pdf]

SUPPORTING INFORMATION FOR:

Zoboli, O., D. Laner, M. Zessner, and H. Rechberger. 2015. Added values of time series in MFA: The Austrian phosphorus budget from 1990 to 2011. *Journal of Industrial Ecology.*

Summary

This supporting information provides descriptions of flows, stocks, transfer coefficients and equations for their calculation, and data sources.

Table S2-1: Description of flows, equations and data sources for their calculation

Flow N.	Flow name	Description	Calculation	Data sources – goods and readily available flows	Data sources – P concentrations and other calculation factors
F1.1	Import live animals	Amount of P in imported live animals	Yearly imported animal carcass weight x conversion factor of carcass-live weight x PC	Statistik Austria,1	UBA,4<br>Glenck et al. 1995<br>LFL, 2013<br>Sibbesen and Runge-Metzger,1995
F1.2	Export live animals	Amount of P in exported live animals	Yearly imported animal carcass weight x conversion factor of carcass-live weight x PC	Statistik Austria,1	UBA,4<br>Glenck et al. 1995<br>LFL, 2013<br>Sibbesen and Runge-Metzger,1995
F1.3	Manure applied to fields	Amount of P in manure applied on agricultural fields	(Yearly n° of animals x specific P excretion) - (yearly manure treated in biogas plants x PC)	BMLFUW,1<br>E-Control<br>UBA,4<br>Resch et al. 2004<br>Jyväskylä Innovation Oy,2009<br>Pötsch, 2004	Kroiss et al., 1998<br>BMLFUW,4,5
F1.4	Animal products	Amount of P in domestic production of meat, milk and eggs	STAN – principle of mass balance		
F1.5	Fallen stock	Amount of P in animals that died for reasons other than slaughtering	Yearly fallen animal live weight x PC	BMG,1,2<br>UBA,4<br>BMLFUW,2	UBA,4<br>Glenck et al. 1995<br>LFL, 2013<br>Sibbesen and Runge-Metzger,1995
F1.6	Manure to biogas	Amount of P in manure digested in biogas plants	Yearly manure x PC	E-Control<br>UBA,4<br>Resch et al. 2004<br>Jyväskylä Innovation Oy,2009<br>Pötsch, 2004	Kroiss et al., 1998<br>BMLFUW,4,5
F2.1	Atm. deposition agric.	Amount of P transported onto agricultural soils through wet and dry atmospheric deposition	Yearly n° of agricultural hectares x P atmospheric deposition per hectare	BMLFUW,1	Glenck et al., 1995
F2.2	Erosion to forestry	Amount of P eroded from agricultural fields and transported to forestry and miscellaneous soils	Estimation		
F2.3	Agricultural emissions	Amount of P transported from agricultural fields to water bodies	MONERIS model results for 2001-2006, yearly scaled through yearly precipitation factor	Zessner et al., 2011	
F2.4	Agricultural products	Amount of P in crops supplied to food-animal feed industry	(Yearly crops production x PC) – (Yearly use of energy crops x PC)	Statistik Austria,4-17	LFL, 2013<br>Kroiss et al., 1998
F2.5	Crops to biogas	Amount of P in energy crops used in biogas plants	Yearly consumption of energy crops by biogas plants x PC	E-Control<br>Resch et al. 2004<br>Jyväskylä Innovation Oy,2009<br>Pötsch, 2004	LFL, 2013<br>Kroiss et al., 1998
F2.6	Non marketable feed	Amount of P in non marketable animal feed	Yearly available fodder x PC	BMLFUW,1<br>Statistik Austria,19	COMIFER,2006<br>LFL, 2013<br>Kroiss et al., 1998
F2.7	Crops to biofuels	Amount of P in inland produced crops used for biofuels production	(Yearly consumption of energy crops for biofuels production x PC) x domestic fraction	UBA,5<br>BMLFUW,1	COMIFER,2006<br>European Standard EN14214/EN15487<br>Kroiss et al., 1998<br>Hein and Leemans,2012<br>LFL,2013<br>Rutkowski,1971<br>ORNL

Flow N.	Flow name	Description	Calculation	Data sources – goods and readily available flows	Data sources – P concentrations and other calculation factors
F3.1	Import wood and paper	Amount of P in imported wood and paper	Yearly imported wood and paper x PC	Statistik Austria,18	Kroiss et al., 1998<br>Binder et al., 2009<br>Antikainen et al., 2004
F3.2	Export wood and paper	Amount of P in exported wood and paper	Yearly exported wood and paper x PC	Statistik Austria,18	Kroiss et al., 1998<br>Binder et al., 2009<br>Antikainen et al., 2004
F3.3	Atm. deposition forestry	Amount of P transported onto forestry and miscellaneous soils through wet and dry atmospheric deposition	Yearly n° of forestry and miscellaneous hectares x P atmospheric deposition per hectare	BMLFUW,1	Glenck et al., 1995
F3.4	Pulp industry WW	Amount of P in wastewater of the pulp industry which is treated in-situ	Readily available data	ICPDR,1<br>Kroiss et al.,1998	
F3.5	Forestry emissions	Amount of P transported from forestry and miscellaneous soils to water bodies	MONERIS model results for 2001-2006, yearly scaled through yearly precipitation factor	Zessner et al., 2011	
F3.6	Wood and paper to consumers	Amount of P in wood an paper consumed by households and similar establishments	(Household wood consumption x PC) + (Total paper consumption x PC x fraction consumed by households)	Austrian Energy Agency<br>Austropapier	Kroiss et al., 1998<br>Binder et al., 2009<br>Antikainen et al., 2004
F3.7	Wood & paper to industry	Amount of P in wood and paper consumed by the industrial sector	(Industrial wood consumption x PC) + (Total paper consumption x PC x fraction consumed by industriess)	Austrian Energy Agency<br>Austropapier	Kroiss et al., 1998<br>Binder et al., 2009<br>Antikainen et al., 2004
F3.8	Energy wood	Amount of P in energy wood consumed in biomass plants	Energy wood consumption x PC	BMLFUW,1<br>Austrian Energy Agency	Kroiss et al., 1998<br>Binder et al., 2009<br>Antikainen et al., 2004
F4.1	Import chemicals	Amount of P in imported chemical products	(Yearly imported selected chemical products x PC) – (yearly phosphoric acids used by fertilizer industry x PC)	Statistik Austria,18	Calculations based on molar mass of compounds
F4.2	Import min.fertilizers and phosphate ore	Amount of P in imported phosphate ore and phosphate mineral fertilizers	Yearly imported mineral phosphate products + yearly imported phosphate ore and intermediate products (estimated as difference between domestic production and import of products)	IFA	
F4.3	Import food	Amount of P in imported food	Yearly imported food x PC	Statistik Austria,1-18	LFL, 2013<br>Kroiss et al., 1998
F4.4	Import feed	Amount of P in imported animal feed	Yearly imported animal feed x PC	Statistik Austria,18<br>BMLFUW,1	LFL, 2013<br>Binder et al., 2009
F4.5	Seeds	Amount of P in seeds	Yearly crop seeds sales x PC	Statistik Austria,4,7-9<br>BMLFUW,7	White,2012
F4.6	Mineral fertilizers to agriculture	Amount of P in phosphate fertilizers applied in agriculture	Total yearly domestic consumption of phosphate mineral fertilizers x proportion sold for agricultural use	IFA<br>BMLFUW,1	Heinzlmaier, 2010<br>Strasser,2010
F4.7	Export feed	Amount of P in exported animal feed	Yearly exported animal feed x PC	Statistik Austria,18<br>BMLFUW,1	LFL, 2013<br>Kroiss et al., 1998<br>Binder et al., 2009
F4.8	Export food	Amount of P in exported food	Yearly exported food x PC	Statistik Austria,1-18	LFL, 2013<br>Kroiss et al., 1998
F4.9	Export mineral fertilizers	Amount of P in exported phosphate fertilizers	Yearly exported phosphate mineral fertilizers	IFA	
F4.10	Export chemicals	Amount of P in exported chemical products	Yearly exported selected chemical products x PC	Statistik Austria,18	Calculations based on molar mass of compounds
F4.11	Municipal ind. WW	Industrial contribution to P load in municipal wastewater	Total yearly P load in municipal wastewater – P load in households municipal wastewater	Statistik Austria,22<br>BMLFUW,8,9	Lindtner and Zessner, 2003<br>Posch, 1999

Flow N.	Flow name	Description	Calculation	Data sources – goods and readily available flows	Data sources – P concentrations and other calculation factors
F4.12	Detergents	Amount of P in detergents used in households and similar establishments	Yearly n° of inhabitants x specific P detergents consumption	Binder et al.,2009<br>de Madariaga et al.,2007<br>Statistik Austria,22	
F4.13	Min. fertilizers to consumers	Amount of P in phosphate fertilizers applied in private gardens and urban green areas	Yearly total domestic consumption of phosphate mineral fertilizers x proportion sold for uses other than agriculture	IFA<br>BMLFUW,1	Heinzlmaier, 2010<br>Strasser,2010
F4.14	Pet food	Amount of P consumed through pet food	Yearly n° of cats and dogs x average P consumption per animal	IEMT	Kalmykova, 2012
F4.15	Food	Amount of P in food consumed by population	Yearly available food supply x PC	Statistik Austria,1-17<br>BMLFUW,6	LFL, 2013<br>Kroiss et al., 1998
F4.16	Other industrial waste	Amount of P in wood and paper waste generated by industrial sector	Yearly industrial wood and paper waste x PC	BMLFUW,2	Kroiss et al., 1998<br>Binder et al., 2009<br>Antikainen et al., 2004
F4.17	Vegetal industrial waste	Amount of P in vegetal wastes and by-products generated by the industry	(Yearly industrial waste of vegetal origin x PC) – (yearly industrial waste of vegetal origin directly reused for animal feed production x PC)	BMLFUW,2<br>Statistik Austria	LFL, 2013<br>Kroiss et al., 1998<br>Binder et al., 2009<br>Klages et al., 2009
F4.18	Animal industrial waste	Amount of P in animal wastes and by-products generated by the industry	(Yearly industrial waste of animal origin x PC) – (yearly industrial waste of animal origin directly reused for animal feed production x PC)	BMLFUW,2<br>UBA,4	Binder et al., 2009<br>Hoppenheidt et al. 2008<br>Kroiss et al., 1998<br>Lamprecht et al., 2011<br>LFL, 2013
F4.19	Marketable feed	Amount of P in marketable animal feed consumption	Yearly available marketable animal feed x PC	BMLFUW,1<br>Statistik Austria,19	LFL, 2013<br>Kroiss et al., 1998<br>Binder et al., 2009
F4.20	In situ ind. WW	Amount of P in industrial wastewater treated in situ	Readily available data	BMLFUW,8,9<br>Kroiss et al.,1998<br>ICPDR,1	
F5.1	Biomass ashes to landscaping	Amount of P in biomass ashes applied in landscaping activities	(Yearly total biomass ashes – yearly landfilled biomass ashes) x proportion applied in landscaping activities x PC	Austrian Energy Agency<br>UBA,2,8	Obernberger and Supancic, 2009
F5.2	Biomass ashes to fields	Amount of P in biomass ashes applied on agricultural fields	(Yearly of total biomass ashes – yearly landfilled biomass ashes) x proportion applied in agriculture x PC	Austrian Energy Agency<br>UBA,2,8	Obernberger and Supancic, 2009
F5.3	Biomass ashes to green areas	Amount of P in biomass ashes applied on private gardens and public green areas	(Yearly total biomass ashes – yearly landfilled biomass ashes) x proportion applied in private gardens and public green areas x PC	Austrian Energy Agency<br>UBA,2,8	Obernberger and Supancic, 2009
F5.4	Biogas digestates	Amount of P in digestates generated by biogas plants	STAN – Principle of mass conservation		
F5.5	Landfilled biomass ashes	Amount of P in biomass ashes disposed of in landfills	Yearly landfilled biomass ashes x PC	Austrian Energy Agency<br>UBA,2,8	Obernberger and Supancic, 2009
F5.6	Biofuels by-products as feed	Amount of P in by-products generated by the biofuel industry	(Yearly generation of bioethanol by-products x PC) + (yearly production of biodiesel x ratio by-product/product x PC)	UBA,5<br>BMLFUW,1	COMIFER,2006<br>European Standard EN14214/EN15487<br>Kroiss et al., 1998<br>Hein and Leemans,2012<br>LFL,2013<br>Rutkowski,1971<br>Simpson et al.,2008<br>ORNL

Flow N.	Flow name	Description	Calculation	Data sources – goods and readily available flows	Data sources – P concentrations and other calculation factors
F5.7	Import raw materials for biofuels	Amount of P in imported raw materials for biofuel production	(Yearly consumption of energy crops for biofuels production x PC) x imported fraction	UBA,5<br>BMLFUW,1	COMIFER,2006<br>European Standard EN14214/EN15487<br>Kroiss et al., 1998<br>Hein and Leemans,2012<br>LFL,2013<br>Rutkowski,1971<br>ORNL
F6.1	Municipal households WW	Households municipal wastewater	(Yearly n° of inhabitants connected to sewers x yearly specific P load per capita in wastewater) + (Yearly fecal sludge treated in MWWTP x PC)	Statistik Austria,22<br>BMLFUW,8,9	Posch, 1999<br>Lindtner and Zessner, 2003<br>Kroiss et al., 2008
F6.2	Residual waste	Amount of P in residual waste from households and similar establishments	Yearly household residual waste x PC (PC yearly modified according to proportion of organic fraction)	BMLFUW,2	Glenck et al., 1995<br>Skutan and Brunner, 2006
F6.3	Separate org. waste	Amount of P in separately collected organic waste from households and similar establishments	Yearly separately collected organic waste x PC	BMLFUW,2	EPEA, 2008<br>Glenck et al., 1995<br>Sokka et al., 2004
F6.4	Green waste	Amount of P in separately collected green waste from private gardens and public green areas	Yearly separately collected green waste x PC	BMLFUW,2	Binder et al., 2009<br>Kroiss et al., 1998
F6.5	Waste wood and paper	Amount of P in separately collected wood and paper waste from households and similar establishments	Yearly separately collected wood and paper waste x PC	BMLFUW,2	Antikainen et al., 2004<br>Binder et al., 2009<br>Kroiss et al., 1998
F6.6	Fecal sludge to groundwater	Amount of P in fecal sludge discharged in the underground and groundwater	Readily available data as of 1992, yearly scaled according to connection rate of the population to the sewer system and the fraction of fecal sludge treated in MWWTP	BMLFUW,8,9<br>Kroiss et al., 1998	
F6.7	Fecal sludge to agriculture	Amount of P in fecal sludge directly applied on agricultural fields	Readily available data as of 1992, yearly scaled according to connection rate of the population to the sewer system and the fraction of fecal sludge treated in MWWTP	BMLFUW,8,9<br>Kroiss et al., 1998	
F7.1	Sewage sludge	Amount of P contained in municipal and industrial sewage sludge	STAN – principle of mass conservation		
F7.2	WW effluents	Amount of P contained in effluent of municipal and industrial WWTP	STAN – principle of mass conservation		
F7.3	Stormwater overflow	Amount of P contained in stormwater overflow	3% of P load of municipal wastewater	BMLFUW,8,9<br>Fenz, 2002	
F8.1	Compost to consumers	Amount of P in compost applied on private gardens and public green areas	Total compost production x PC x fraction used in private gardens and public green areas	BMLFUW,2,3	BMLFUW,3
F8.2	M&B meal to animal feed	Amount of P in meat and bone meal used as animal feed	Meat and bone meal reused as animal feed x PC	BMLFUW,2<br>UBA,4<br>BGBl. I Nr. 143/2000	Binder et al., 2009<br>Kroiss et al., 1998<br>Lamprecht et al., 2011<br>Klock and Taber, 1996
F8.3	Substrate landscaping	Amount of P in wastes recovered for substrate landscaping	STAN – principle of mass conservation		
F8.4	Recycled wood and paper	Amount of P in wood and paper waste that is recycled	STAN – principle of mass conservation		
F8.5	Wastes recycled in agriculture	Amount of P in wastes and by-products applied on agricultural fields	STAN – principle of mass conservation		
F8.6	Export sewage sludge	Amount of P in exported sewage sludge	Total yearly P in sewage sludge x yearly exported fraction	BMLFUW,1,8,9<br>UBA,1,6,7	

Flow N.	Flow name	Description	Calculation	Data sources – goods and readily available flows	Data sources – P concentrations and other calculation factors
F8.7	Export M&B meal	Amount of P in exported meat and bone meal	Yearly exported meat and bone meal x PC	BMLFUW,2<br>UBA,4	Binder et al., 2009<br>Kroiss et al., 1998<br>Lamprecht et al., 2011<br>Klock and Taber, 1996
F8.8	Export filter cakes	Amount of P in exported filter cakes	STAN – principle of mass conservation		
F8.9	Export organic waste	Amount of P in exported organic waste	Yearly exported organic waste x PC	BMLFUW,2<br>UBA,4	Binder et al., 2009<br>Klages et al., 2009<br>Kroiss et al., 1998<br>LFL, 2013
F8.10	Waste to biogas plants	Amount of P in industrial wastes and by-products treated in biogas plant	STAN – principle of mass conservation		
F8.11	Green waste to biomass plants	Amount of P in green waste used in biomass thermal plants	Yearly generation of green waste x PC x proportion used to biomass thermal plants	BMLFUW,2	Binder et al., 2009<br>Kroiss et al., 1998
F8.12	Import animal waste	Amount of P in imported animal waste	Yearly imported animal waste x PC	BMLFUW,2<br>UBA,4	Hoppenheidt et al.,2000<br>Kroiss et al., 1998<br>Lamprecht et al., 011<br>LFL,2013
F9.1	Import water bodies	Amount of P in rivers at their entrance into Austria	Modeling of P yearly load based on daily water flow and monthly P concentration	BMLFUW, 10<br>ICPDR,2	BMLFUW, 11<br>ICPDR,2
F9.2	Export water bodies	Amount of P in rivers at their exit from Austria	Modeling of P yearly load based on daily water flow and monthly P concentration	BMLFUW, 10<br>ICPDR,2	BMLFUW, 11<br>ICPDR,2
P1.1	Meat production	Amount of P in domestic meat production	Yearly meat production x PC	Statistik Austria,1<br>BMLFUW,1	Kroiss et al., 1998<br>LFL, 2013
P1.2	Eggs and milk production	Amount of P in domestic eggs and milk production	(Yearly eggs production x PC) + (Yearly milk production x PC)	Statistik Austria,2,3<br>BMLFUW,1	Kroiss et al., 1998<br>LFL, 2013
P3.1	Timber	Amount of P in domestic timber production	Yearly timber extraction x PC	Statistik Austria,21<br>BMLFUW,1<br>Waldinventur	Kroiss et al., 1998<br>Antikainen et al., 2004
P4.1	Products to food production	Amount of P in domestic crops allocated to food production	STAN – principle of mass conservation		
P4.2	Products to feed production	Amount of P in domestic crops allocated to marketable animal feed production	STAN – principle of mass conservation		
P4.3	By-products to animal feed production	Amount of P in food industry by-products directly reused for animal feed production	Yearly organic by-products of the food industry directly reused for animal feed production x PC	Statistik Austria,20<br>UBA,4	LFL, 2013<br>Kroiss et al., 1998<br>Binder et al., 2009
P4.4	Food WW	Amount of P in food industry wastewater connected to municipal sewer	P load in industrial wastewater treated in municipal WWTP x fraction corresponding to food industry	BMLFUW,8,9<br>Kroiss et al.,1998<br>ICPDR,1	
P4.5	Animal feed WW	Amount of P in feed industry wastewater connected to municipal sewer	P load in industrial wastewater treated in municipal WWTP x fraction corresponding to feed industry	BMLFUW,8,9<br>Kroiss et al.,1998<br>ICPDR,1	
P4.6	Fertilizer WW	Amount of P in fertilizer industry wastewater connected to municipal sewer	P load in industrial wastewater treated in municipal WWTP x fraction corresponding to fertilizer industry	BMLFUW,8,9<br>Kroiss et al.,1998<br>ICPDR,1	
P4.7	Chemical WW	Amount of P in chemical industry wastewater connected to municipal sewer	P load in industrial wastewater treated in municipal WWTP x fraction corresponding to chemical industry	BMLFUW,8,9<br>Kroiss et al.,1998<br>ICPDR,1	
P4.8	Fertilizer industry in situ WW	Amount of P in fertilizer industry wastewater treated in situ	P load in wastewater of chemical and fertilizer industry treated in situ x fraction corresponding to fertilizer industry	BMLFUW,8,9<br>Kroiss et al.,1998<br>ICPDR,1	

Flow N.	Flow name	Description	Calculation	Data sources – goods and readily available flows	Data sources – P concentrations and other calculation factors
P4.9	Chemical industry in situ WW	Amount of P in chemical industry wastewater treated in situ	P load in wastewater of chemical and fertilizer industry treated in situ x fraction corresponding to chemical industry	BMLFUW,8,9<br>Kroiss et al.,1998<br>ICPDR,1	
P6.1	Organic waste to home composting	Amount of P in organic waste from households and similar establishments, handled through home composting	Separately collected household organic waste x PC	BMLFUW,2	EPEA, 2008<br>Glenck et al., 1995<br>Sokka et al., 2004
P6.2	Garden vegetables	Amount of P in vegetables grown in private gardens	Yearly garden vegetables production x PC	Statistik Austria,12	COMIFER,2006<br>Kroiss et al., 1998<br>LFL,2013
P6.3	Garden waste	Amount of P in garden residues collected together with household waste	Yearly separately collected household organic waste x fraction composed of garden waste x PC	BMLFUW,2	Binder et al., 2009<br>Kroiss et al., 1998
P6.4	Domestic animals excretions	Amount of P in domestic animals excretions, ending up on gardens and public green areas	Estimation		
P6.5	Wood & paper in residual waste	Amount of P in wood and paper fraction of household residual waste	Yearly household residual waste x fraction of wood and paper x PC	BMLFUW,2	Kroiss et al., 1998<br>Binder et al., 2009<br>Antikainen et al., 2004
P7.1	Municipal WW to MWWTP	Amount of P in municipal wastewater treated in WWTP	Readily available data	BMLFUW,8,9	
P7.2	Municipal effluent	Amount of P in effluents of municipal WWTP	Readily available data	BMLFUW,8,9	
P7.3	Municipal sewage sludge	Amount of P in sewage sludge generated by municipal WWTP	Input – output in municipal WWTP (both readily available data)	BMLFUW,2,8,9<br>UBA,1,6,7	
P7.4	Industrial effluent	Amount of P in effluents of industrial in situ WWTP	Readily available data	BMLFUW,8,9	
P7.5	Industrial sewage sludge	Amount of P in sewage sludge generated by industrial in situ WWTP	Input – output in industrial in situ WWTP (both readily available data)	BMLFUW,8,9<br>Kroiss et al., 1998<br>UBA,1	
P8.1	Animal waste to Rendering	Amount of P in animal waste and by-products handled through the rendering process	Yearly animal waste and by-products handled through rendering x PC	BMLFUW,2<br>UBA,4	Lamprecht, 2011<br>Kroiss, 1998<br>EU, 2001
P8.2	Green waste to compost	Amount of P in green wastes handled through composting process	Yearly separately collected green wastes handled through composting x PC	BMLFUW,2,3	BMLFUW,3<br>Binder et al., 2009<br>Kroiss et al., 1998
P8.3	Animal waste to biogas	Amount of P in animal waste handled in biogas plants	Yearly animal waste and by-products used in biogas plants x PC	E-Control<br>UBA,4<br>Resch et al. 2004<br>Jyväskylä Innovation Oy,2009<br>Pötsch, 2004	Lamprecht, 2011<br>Kroiss, 1998<br>EU, 2001
P8.4	HH res. waste to MBT	Amount of P in household residual waste handled through mechanical-biological treatment	Yearly household residual waste x PC (PC yearly modified according to proportion of organic fraction) x fraction stabilized through mechanical-biological treatment	BMLFUW,2<br>UBA,3	Glenck et al., 1995<br>Skutan and Brunner, 2006
P8.5	HH res. waste to landfill	Amount of P in landfilled household residual waste	Yearly household residual waste x PC (PC yearly modified according to proportion of organic fraction) x landfilled fraction	BMLFUW,2	Glenck et al., 1995<br>Skutan and Brunner, 2006
P8.6	HH res. waste to TT	Amount of P in incinerated household residual waste handled	Yearly household residual waste x PC (PC yearly modified according to proportion of organic fraction) x incinerated fraction	BMLFUW,2	Glenck et al., 1995<br>Skutan and Brunner, 2006
P8.7	SS to composting	Amount of P in composted sewage sludge	Total yearly P in sewage sludge x yearly composted fraction	BMLFUW,2,3,8,9<br>UBA,1,6,7	

Flow N.	Flow name	Description	Calculation	Data sources – goods and readily available flows	Data sources – P concentrations and other calculation factors
P8.8	SS to landfill	Amount of P in landfilled sewage sludge	Total yearly P in sewage sludge x yearly landfilled fraction	BMLFUW,2,8,9<br>UBA,1,6,7	
P8.9	SS to TT	Amount of P in incinerated sewage sludge	Total yearly P in sewage sludge x yearly incinerated fraction	BMLFUW,2,8,9<br>UBA,1,6,7	
P8.10	SS to co-incineration	Amount of P in sewage sludge incinerated in specific sludge co-incineration plants	Total yearly P in sewage sludge x yearly fraction incinerated in sludge co-incineration plants	BMLFUW,2,8,9<br>UBA,1,6,7	
P8.11	SS applied in agriculture	Amount of P in sewage sludge directly applied on agricultural fields	Total yearly P in sewage sludge x yearly fraction directly applied in agriculture	BMLFUW,2,8,9<br>UBA,1,6,7	
P8.12	SS to landscaping	Amount of P in sewage sludge applied in landscaping activities	Total yearly P in sewage sludge x yearly fraction applied in landscaping activities	BMLFUW,2,8,9<br>UBA,1,6,7	
P8.13	M&B meal as fertilizer	Amount of P in meat and bone meal applied on agricultural fields	Yearly meat and bone meal applied on agricultural fields x PC	BMLFUW,2<br>UBA,4	Binder et al., 2009<br>Kroiss et al., 1998<br>Lamprecht et al., 2011<br>Klock and Taber, 1996
P8.14	M&B meal to fuel	Amount of P in meat and bone meal used as fuel in cement kilns	Yearly meat and bone meal used as fuel in cement kilns x PC	BMLFUW,2<br>UBA,4	Binder et al., 2009<br>Kroiss et al., 1998<br>Lamprecht et al., 2011<br>Klock and Taber, 1996
P8.15	Stabilized waste to landfill	Amount of P in waste stabilized through mechanical-biological treatment and then landfilled	Yearly waste stabilized through mechanical-biological treatment and then landfilled x PC	BMLFUW,2<br>UBA,3	Estimation
P8.16	Compost to agriculture	Amount of P in compost product applied on agricultural fields	Total compost production x PC x fraction applied on agricultural fields	BMLFUW,2,3	BMLFUW,3
P8.17	Compost to landscaping	Amount of P in compost product applied in landscaping activities	Total compost production x PC x fraction applied in landscaping activities	BMLFUW,2,3	BMLFUW,3
P8.18	Stabilized waste to TT	Amount of P in waste stabilized through mechanical-biological treatment and then incinerated	Yearly waste stabilized through mechanical-biological treatment and then incinerated x PC	BMLFUW<br>UBA,3	Estimation
P8.19	Ash co-inc. to landfill	Amount of P in landfilled ashes of sludge co-incineration	STAN – transfer coefficient		
P8.20	Ash/slag to landfill	Amount of P in landfilled ashes of municipal waste incineration	STAN – transfer coefficient		
P8.21	Loss in clinker	Amount of total P lost in cement kilns	STAN – principle of mass conservation		
P8.22	SS to fuel	Amount of P in sewage sludge used as fuel in cement kilns	Total yearly P in sewage sludge x yearly fraction used as fuel in cement kilns	BMLFUW,2,8,9<br>UBA,1,6,7	
P8.23	SS to waste incineration	Amount of P in sewage sludge incinerated in municipal waste incineration	Total yearly P in sewage sludge x yearly fraction incinerated with municipal waste	BMLFUW,2,8,9<br>UBA,1,6,7	
P8.24	Filter cakes Inc.	Amount of P in filter cakes generated by municipal waste incineration	STAN – transfer coefficient		
P8.25	Filter cakes Co-inc.	Amount of P in filter cakes generated by sludge co-incineration	STAN – transfer coefficient		
P8.26	SS to MBT	Amount of P in sewage sludge stabilized through mechanical-biological process	Total yearly P in sewage sludge x yearly fraction stabilized through mechanical-biological treatment	BMLFUW,2,8,9<br>UBA,1,3,6,7	
P8.27	Animal waste to compost	Amount of P in composted waste and by-products of animal origin	Yearly composted animal waste and by-products x PC	BMLFUW,2,3<br>UBA,4	BMLFUW,3
P8.28	Veg. ind. to biogas	Amount of P in organic industrial waste of vegetal source treated in biogas plants	Industrial waste of vegetal source used by biogas plants x PC	E-Control<br>Resch et al. 2004<br>Jyväskylä Innovation Oy,2009<br>Pötsch, 2004	LFL, 2013<br>Kroiss et al., 1998<br>Binder et al., 2009

Table S2-2: Description of stocks and transfer coefficients, equations and data sources for their calculation

Stock N.	Stock name	Description	Calculation	Data sources – goods and readily available flows	Data sources – P concentrations and other calculation factors
S1	Livestock	Amount of P in the livestock at the beginning of the year	Yearly livestock x PC	BMLFUW,1	Kroiss et al., 1998<br>LFL, 2013
S2	Agricultural fields	Amount of P in the agicultural fields at the beginning of the year	Estimation		
S3	Trees and soil	Amount of P in the trees, forestry and miscellaneous soils at the beginning of the year	Estimation		
S6-1	Stock in buildings and furniture	Amount of P in the buildings and furniture at the beginning of the year			
S6-2	Private gardens & public green areas	Amount of P in the soils o private gardens and public green areas at the beginning of the year	Estimation		
S8-1	Clinker	Amount of P lost in the cement kilns at the beginning of the year	Estimation of loss until beginning of 1990		
S8-2	Landfills	Amount of P in the landfills at the beginning of the year			
S9	Water bodies	Amount of P in the water bodies at the beginning of the year			

Stock N.	Stock change rate name	Description	Calculation	Data sources – goods and readily available flows	Data sources – P concentrations and other calculation factors
S1	Livestock	Yearly change of P in the livestock at the beginning of the year	(Yearly livestock x PC) - (Previous year livestock x PC)	BMLFUW,1	Kroiss et al., 1998<br>LFL, 2013
S2	Agricultural fields	Yearly change of P in the agicultural fields at the beginning of the year	STAN – principle of mass conservation		
S3	Trees and soil	Yearly change of P in the trees, forestry and miscellaneous soils at the beginning of the year	STAN – principle of mass conservation		
S6-1	Stock in buildings and furniture	Yearly change of P in the buildings and furniture at the beginning of the year	STAN – principle of mass conservation		
S6-2	Private gardens & public green areas	Yearly change of P in the soils o private gardens and public green areas at the beginning of the year	STAN – principle of mass conservation		
S8-1	Clinker	Yearly change of P lost in the cement kilns at the beginning of the year	STAN – principle of mass conservation		
S8-2	Landfills	Yearly change of P in the landfills at the beginning of the year	STAN – principle of mass conservation		
S9	Water bodies	Yearly change of P in the water bodies at the beginning of the year	STAN – principle of mass conservation		

Process N.	Process name (transfer coefficient)	Description	Calculation	Data sources – goods and readily available flows	Data sources – P concentrations and other calculation factors
P30	Waste incineration	Transfer of phosphorus to ashes during waste incineration		Morf et al., 2005	
P29	Sludge co-incineration	Transfer of phosphorus to ashes during sludge co-incineration		UBA,1	

DATA SOURCES

Antikainen R., R. Haapanen and S. Rekolainen. 2004. Flows of nitrogen and phosphorus in Finland—the forest industry and use of wood fuels; Journal of Cleaner Production

Austrian Energy Agency. Holzströme in Österreich, 2009, 2010, 2011 (German)

Austropapier (Association of the Austrian Paper Industry). Jahresbericht 2010, 2013 (German)

Binder C., L. De Baan and D. Wittmer. 2009. Phosphorflüsse in der Schweiz, Stand, Risiken und Handlungsoptionen, Final report, Umwelt-Wissen Nr. 0928. Bern, German: Bundesamt für Umwelt (German)

BGBl. I Nr. 143/2000 Bundesgesetz zur Umsetzung der Entscheidung des Rates über Schutzmaßnahmen in Bezug auf die transmissiblen spongiformen Enzephalopathien und die Verfütterung von tierischem Protein vom 4. Dezember 2000 (Tiermehl-Gesetz) (German)

BMG (Bundesministerium für Gesundheit Familie und Jugend)
[1] Veterinährjahresbericht 1998,1999,2000,2001,2002,2003,2004,2005,2006 (German)
[2] Personal communication, 2013

BMLFUW (Bundesministerium für Land- und Forstwirtschaft, Umwelt und Wasserwirtschaft) - Lebensministerium
[1] Grüner Bericht 1991, 1992, 1993, 1994, 1995, 1996, 1997, 1998, 1999, 2000, 2001, 2002, 2004, 2005, 2006, 2007, 2008, 2009, 2010, 2011, 2012 (German)
[2] Bundesabfallwirtschaftsplan (BAWP) 1995, 1998, 2001, 2006, 2011 (German)
[3] Stand der Technik der Kompostierung, Wien 2005 (German)
[4] Richtlinien für eine sachgerechte Düngung, 2006 (German)
[5] ÖPUL 2000, Sonderrichtlinie für das Österreichische Programm zur Förderung einer umweltgerechten, extensiven und den natürlichen Lebensraum schützenden Landwirtschaft. Anhang 15. Wien, 2000 (German)
[6] Lebensmittelbericht Österreich. 1995, 1995-2002, 2008, 2010. Wien (German)
[8] Gewässerschutzbericht 1993, 1996, 1999, 2002 (German)
[9] Kommunale Abwasserrichtlinie der EU – 91/271/EWG. Österreichischer Bericht 2008, 2010, 2012 (German)
[10] Wasser Informationsystem WISA. eHYD Hydrographische Daten. http://ehyd.gv.at (German)
[11] Wasser Informationsystem WISA. H2O Fachdatenbank**.** http://wisa.bmlfuw.gv.at/ (German)

COMIFER (Comité Français d'Étude et de Développement de la Fertilisation Raisonneé). 2007. Teneur en P, K et Mg des organes végétaux récoltés pour les cultures de plein champ et les principaux fourrages, Paris (French)

de Madariaga B.M., M.J. Ramos and J.V. Tarazona. 2007. Model implementation and quantification of the eutrophication risk associated to the use of phosphates in detergents. Green Planet Research Report GPR-CEEP-07-1- Expanded Final Report

E-Control, Ökostrombericht. Bericht der Energie-Control GmbH gemäß § 25 Abs 1 Ökostromgesetz. Wien, German: Energie-Control GmbH; 2005, 2006, 2007, 2008, 2009, 2010, 2011, 2012

EPEA (Internationale Umweltforschung GmbH). 2008. Ökologisches Leistungsprofil von Verfahren zur Behandlung von biogenen Reststoffen. Hamburg (German)

European Commission. 2001. Survey of wastes spread on land – final report

European Standard EN 14214:2008. Automotive fuels - Fatty acid methyl esters (FAME) for diesel engines - Requirements and test methods

European Standard EN 15487:2007. Ethanol as a blending component for petrol — Determination of phosphorus content — Ammonium molybdate spectrometric method

Fenz R. 2002. Gewässerschutz bei Entlastungsbauwerken der Mischkanalisation. Wiener Mitteilungen. Band 174, Wien (German)

Glenck, E., C. Lampert, H. Raeissi H and P.H. Brunner. 1995. Phosphorbilanz des Kremstales, Bericht des Institutes für Wassergüte und Abfallwirtschaft im Auftrag des Amtes der Oö. Landesregierung, Wien (German)

Hein L. and R. Leemans. 2012. The impact of first-generation biofuels on the depletion of the global phosphorus reserve. AMBIO, 41:341–349

Heinzlmaier F. 2010. Personal notification from Franz Heinzlmaier, sales manager of Agro Trade Linz (fertilizers production company)

Hoppenheidt, Co-Vergärung von Bioabfällen und organischen Gewerbeabfällen - Ergebnisse eines großtechnischen Pilotvorhabens,VDI Seminar "Biogene Abfälle/Holz/Klärschlamm - Verwertung/Behandlung/Beseitigung -" 13.4.-15.4.2000, Bamberg (German)

ICPDR (International Commission for the Protection of the Danube River)
[1] Danube River Basin District Management Plan 2009
[2] Water Quality Database. http://www.icpdr.org/main/publications/databases

IEMT (Institut für interdisziplinäre Erforschung der Mensch-Tier-Beziehung) http://www.iemt.at/?i_ca_id=386. Accessed on 9.01.2014 (German)

IFA (International Fertilizer Industry Association). Statistics Database 1990-2011. http://ifadata.fertilizer.org/ucSearch.aspx. Accessed on 14.08.2013

Kalmykova Y., R. Harder, H. Borgestedt and I. Svanänga. 2012. Pahtways and management of phosphorus in urban areas, Journal of Industrial Ecology, Volume 16, Number 16

Klages, S., U. Schultheiß and H. Döhler. 2009. Potential and applicability of renewable residual materials and organic waste for fermentation in biogas plants. In Landtechnik 6 S. 398-403

Klock., K.A and H.G. Taber. 1996. Comparison of Bone Products for Phosphorus Availability. Technology & Products Reports 6(3) 257-260

Kroiss H, M. Zessner, K. Deutsch, W. Schaar and N. Kreuzinger. 1998. Nährstoffbilanzen der Donauanrainerstaaten – Erhebungen für Österreich, Studie im Auftrag des österreichischen Bundeskanzleramtes. TU Wien- Institut für Wassergüte und Abfallwirtschaft. (German)

Kroiss, H., L.S. Morf, C. Lampert, M. Zessner and A. Spindler. 2008, Optimiertes Stoffflussmonitoring für die Abwasserentsorgung Wiens OSMA-Wien, Wien (German)

Lamprecht, H., D.J. Lang, C.R. Binder and R.W. Scholz. 2011. The Trade-Off between Phosphorus Recycling and Health Protection during the BSE Crisis in Switzerland. A "Disposal Dilemma", GAIA 20/2: 112–121

LFL, Basisdaten zur Umsetzung der Düngeverordnung, Stand 2013. Bayerische Landesanstalt für Landwirtschaft, http://www.lfl.bayern.de/iab/duengung/031245/ accessed on 15.01.2014 (German)

Lindtner S. and M. Zessner. 2003. Abschätzung von Schmutzfrachten in der Abwasserentsorgung bei unvollständiger Datenlage. In: Kroiss H., editor. Wiener Mitteilungen: Wasser-Abwasser-Gewässer, Band 183. Fortbildungsseminar Abwasserentsorgung. Institut für Wassergüte und Abfallwirtschaft, TU Wien (German)

Morf L.S., Ritter E., and P.H. Brunner. 2005. Online-Messung auf der MVA Spittelau - Endbericht 1.-5. Messjahr, Wien (German)

Obernberger I. and K. Supancic. Possibilities of ash utilisation from biomass combustion plants. Proceedings of the 17th European Biomass Conference & Exhibition, June/July 2009, Hamburg, ETA-Renewable Energies (Ed.), Italy

ORNL (Oak Ridge National Laboratory). https://bioenergy.ornl.gov/papers/misc/energy_conv.html. Accessed on 29.11.2013

Posch, A. 1999. Die Konzepzion kommunaler Abwasserbehandlungsysteme aus ökonomischer Sicht. PhD thesis, Graz (German)

Pötsch, Erich M. 2004. Nährstoffgehalt von Gärrückständen aus landwirtschaftlichen Biogasanlagen und deren Einsatz im Dauergrünland. Endbericht (German)

Resch,R., E.M.Pötsch, and E. Pfuntner. Biogasanlagen in Österreich - ein aktueller Überblick.10. Alpenländisches Expertenforum, 18. - 19. März 2004 (German)

Rutkowski A. 1971. The feed value of rapeseed meal, Journal of the American Oil Chemists Society, Volume 48, Issue 12, pp 863-868

Sibbesen E. and A. Runge-Metzger. 1995. Phosphorus Balance In European Agriculture - Status And Policy Options. Phosphorus in the Global Environment. H. Tiessen (Editor), John Wiley & Sons. Ltd. http://www.scopenvironment.org/downloadpubs/scope54/4sibbesen.htm accessed on 10.12.2013

Simpson T.W., A.N. Sharpley, R.W. Howarth, H.W. Paerl and K.R. Mankin. 2008. The new gold rush: fueling ethanol production while protecting water quality. Journal of Environmental Quality, Volume 37

Skutan S. and P.H. Brunner. 2006. Stoffbilanzen mechanisch-biologischer Anlagen zur Behandlung von Restmüll (SEMBA), Endbericht, Wien (German)

Sokka, L., R. Antikainen R. and P. Kauppi. 2004. Flows of nitrogen and phosphorus in municipal waste: a substance flow analysis in Finland, Progress in Industrial Ecology, Vol. 1, Nos. 1/2/3

Statistics Austria

[1] Supply balance sheet for meat by species as of 1994
[2] Supply balance sheet for raw milk and dairy products as of 1995
[3] Supply balance sheet for eggs as of 1994
[4] Supply balance sheet for cereals as of 1994/1995
[5] Supply balance sheet for fruits as of 1994/1995
[6] Supply balance sheet for honey as of 1994
[7] Supply balance sheet for oil seeds as of 1994/1995
[8] Supply balance sheet for potatoes and potatoes starch as of 1994/1995
[9] Supply balance sheet for pulses as of 1994/1995
[10] Supply balance sheet for sugar as of 1994/1995
[11] Supply balance sheet for vegetable fats and oils as of 1994/1995
[12] Supply balance sheet for vegetables as of 1994/1995
[13] Supply balance sheet for wine as of 1994/1995
[14] Supply balance sheet for beer as of 1994/1995
[15] Supply balance sheet for rice as of 1994/1995
[16] Supply balance sheet for fish as of 1994/1995
[17] Supply balance sheet for animal fats as of 1995
[18] Foreign trade 1995-2011
[19] Futtermittelbilanz; LFZ Raumberg-Gumpenstein. Erstellt am 29.06.2012 (German)
[20] Personal communication, Frau Bader, 2013
[21] Integrierte NAMEA 1995 – 2009 (German)
[22] Population at the beginning of the year since 1982

Strasser M. 2010. Personal notification from Max Strasser, sales manager of Timac-Agro Pischelsdorf (fertilizers production company)

UBA - Umweltbundesamt

[1] Klärschlamm. Materialien zur Abfallwirtschaft. Klagenfurt 2009 (German)
[2] Abfallvermeidung -und Verwertung: Aschen, Schlacken und Stäube in Österreich. Wien 2005 (German)

[3] Ist-Stand der Mechanisch-Biologischen Abfallbehandlung (MBA) in Österreich, Wien 2006 (German)
[4] Tierische Nebenprodukte 2004–2006. Erhebung der Mengen an tierischen Nebenprodukten in Österreich, Wien 2008 (German)
[5] Biokraftstoffe im Verkehrssektor in Österreich Bericht (Biofuels in the transport sector in Austria – Report) 2004, 2005, 2006, 2007, 2008, 2009, 2010, 2011, 2012 (German)
[6] Austria's Informative Report (IIR) 2011. Submission under the UNECE Convention on Long-range Transboundary Air Pollution. Vienna, 2011
[7] Zur Situation der Verwertung und Entsorgung des kommunalen Klärschlammmes in Österreich. Monographien Band 095. Wien, 1997 (German)
[8] Die Bestandsaufnahme der Abfallwirtschaft in Österreich Statusbericht 2012 (German)

Waldinventur. Holznutzung pro Jahr. Erhebung 1992-1996, 2000-2002, 2007-2009. www.waldinventur.at. Accessed on 28.01.2013 (German)

White P.J. and E.J. Veneklaas. 2012. Nature and nurture: the importance of seed phosphorus content, Plant soil 357:1-8

Jyväskylä Innovation Oy. 2009. Energy from field energy crops – a handbook for energy producers. Jyväskylä Innovation Oy & MTT Agrifood Research Finland

Zessner M., A. Kovacs, S. Thaler, G. Hochedlinger, C. Schilling and G. Windhofer. 2008. Optimiertes Stoffflussmonitoring für die Abwasserentsorgung Wiens OSMA-Wien (German)
