# Supplementary material for: Added Values of Time Series in Material Flow Analysis: The Austrian Phosphorus Budget from 1990 to 2011
Source: J Ind Ecol. 2015 Dec 22;20(6):1334–48. doi: 10.1111/jiec.12381 (PMC5217078; doi:10.1111/jiec.12381)
Supplement: Supplementary file 3 — Supporting Information S3: This supporting information characterizes data uncertainty. [file 44498_2016_2006008_MOESM3_ESM.pdf]

SUPPORTING INFORMATION FOR:

Zoboli, O., D. Laner, M. Zessner, and H. Rechberger. 2015. Added values of time series in MFA: The Austrian phosphorus budget from 1990 to 2011. *Journal of Industrial Ecology.*

Summary

This supporting information characterizes data uncertainty.

The CV values presented in Table 2 in the manuscript are dependent only on two parameters for each evaluation score (1 to 4). The parameter values for the different sensitivity levels are defined as follows: $a_{\text{not sensitive}}$= 1.0, $a_{\text{sensitive}}$ = 2.6, $a_{\text{highly sensitive}}$= 4.5, $b_{\text{not sensitive}}$= $b_{\text{sensitive}}$= 0.71, $b_{\text{highly sensitive}}$= 0.8.

For score = 1: CV = 0

For score = [1, 4]: CV = $a \cdot e^{b \cdot (score-1)}$ (1)

The CVs for *Reliability* and *Expert Judgment* are determined using a slightly adapted function (Equation 2), because the best score should not result in zero uncertainty. The parameter values used for *Reliability* are a= 0.02 and b= 0.805, whereas for *Expert Judgment* a=0.05 and b= 0.692. For score= [1, 4]: CV = $a \cdot e^{b \cdot score}$ (2)

The resulting functions are depicted below in Figure 1.

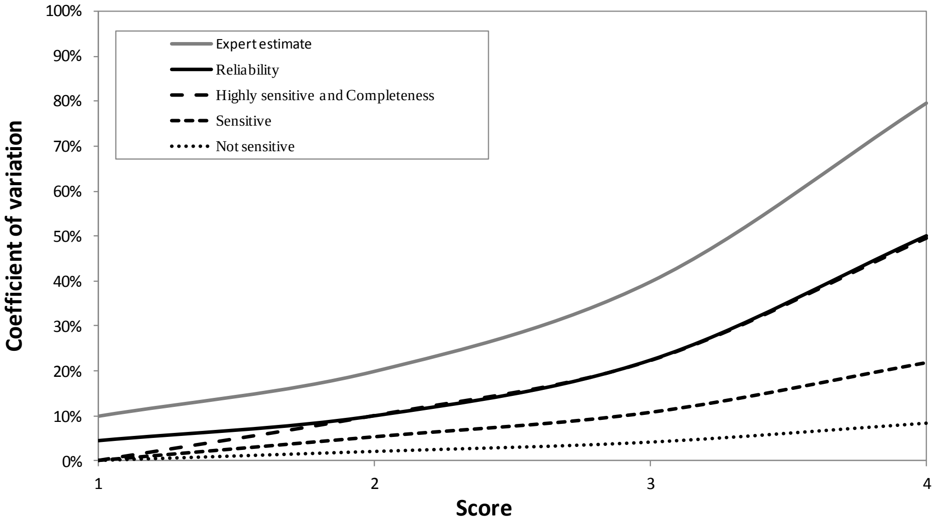

Figure S3- 1: Continous functions that reflect the relationships between indicator scores and coefficients of variation

Table S3-1: Relative uncertainty of the input data for the flows.

Empty lines indicate that no input values were calculated and that they were calculated directly by the software STAN, according to the principle of mass conservation.

Flow N.	Flow name	1990	1991	1992	1993	1994	1995	1996	1997	1998	1999	2000	2001	2002	2003	2004	2005	2006	2007	2008	2009	2010	2011
F1.1	Import live animals	22%	20%	19%	18%	12%	12%	12%	12%	12%	12%	12%	12%	12%	12%	12%	12%	12%	12%	12%	12%	12%	12%
F1.2	Export live animals	22%	20%	19%	18%	12%	12%	12%	12%	12%	12%	12%	12%	12%	12%	12%	12%	12%	12%	12%	12%	12%	12%
F1.3	Manure applied to fields	27%	27%	27%	27%	27%	27%	27%	27%	27%	27%	27%	27%	27%	27%	27%	27%	27%	27%	27%	27%	27%	27%
F1.4	Animal products																						
F1.5	Fallen stock	40%	41%	41%	42%	43%	44%	43%	42%	41%	41%	40%	40%	40%	39%	39%	39%	39%	40%	40%	39%	40%	41%
F1.6	Manure to biogas	61%	61%	61%	55%	50%	47%	44%	41%	40%	38%	38%	37%	36%	35%	36%	37%	37%	35%	35%	35%	37%	37%
F2.1	Atm. deposition agric.	24%	24%	24%	24%	24%	24%	24%	24%	24%	24%	24%	24%	25%	25%	26%	27%	28%	30%	32%	32%	32%	32%
F2.2	Erosion to forestry	80%	80%	80%	80%	80%	80%	80%	80%	80%	80%	80%	80%	80%	80%	80%	80%	80%	80%	80%	80%	80%	80%
F2.3	Agricultural emissions	32%	32%	30%	28%	27%	26%	25%	25%	24%	24%	24%	23%	23%	23%	23%	23%	23%	24%	24%	24%	25%	26%
F2.4	Agricultural products	20%	20%	20%	20%	17%	14%	14%	14%	14%	14%	14%	14%	14%	14%	14%	14%	14%	14%	14%	14%	14%	14%
F2.5	Crops to biogas	55%	55%	55%	49%	43%	39%	35%	32%	30%	29%	27%	26%	26%	24%	26%	35%	34%	33%	33%	33%	34%	35%
F2.6	Non marketable feed	18%	18%	18%	18%	18%	18%	18%	18%	18%	18%	18%	18%	18%	18%	18%	18%	18%	18%	18%	18%	18%	18%
F2.7	Crops to biofuels	12%	12%	12%	12%	12%	12%	12%	12%	12%	12%	12%	12%	21%	41%	41%	41%	41%	41%	41%	41%	41%	41%
F3.1	Import wood and paper	27%	27%	27%	26%	25%	25%	25%	25%	25%	25%	25%	25%	25%	25%	24%	20%	20%	21%	20%	20%	20%	20%
F3.2	Export wood and paper	27%	27%	27%	26%	25%	25%	25%	25%	25%	25%	25%	25%	25%	25%	24%	25%	25%	25%	25%	25%	25%	25%
F3.3	Atm. deposition forestry	24%	24%	24%	24%	24%	24%	24%	24%	24%	24%	24%	24%	25%	25%	26%	27%	28%	30%	32%	32%	32%	32%
F3.4	Pulp industry WW	23%	23%	23%	23%	20%	18%	16%	14%	13%	12%	11%	10%	9%	9%	7%	9%	9%	10%	11%	12%	13%	14%
F3.5	Forestry emissions	32%	32%	30%	28%	27%	26%	25%	25%	24%	24%	24%	23%	23%	23%	23%	23%	23%	24%	24%	24%	25%	26%
F3.6	Wood and paper to consumers	30%	30%	30%	30%	30%	28%	26%	25%	23%	22%	22%	21%	21%	20%	20%	20%	20%	21%	20%	20%	20%	20%
F3.7	Wood & paper to industry	30%	30%	30%	30%	30%	28%	26%	25%	23%	22%	22%	21%	21%	20%	20%	20%	20%	21%	20%	20%	20%	20%
F3.8	Energy wood	29%	29%	29%	29%	28%	26%	24%	22%	21%	20%	19%	19%	18%	18%	17%	17%	18%	18%	18%	17%	17%	17%
F4.1	Import chemicals	25%	22%	21%	19%	18%	15%	15%	15%	15%	15%	15%	15%	15%	15%	15%	15%	15%	15%	15%	15%	15%	15%
F4.2	Import min.fertilizers and phosphate ore	25%	25%	25%	25%	25%	25%	25%	25%	25%	25%	25%	25%	25%	25%	25%	25%	25%	25%	25%	25%	25%	25%
F4.3	Import food	23%	21%	20%	19%	13%	13%	13%	13%	13%	13%	13%	13%	13%	12%	12%	11%	11%	11%	11%	10%	10%	10%
F4.4	Import feed	23%	23%	23%	23%	24%	12%	12%	12%	12%	12%	12%	12%	12%	12%	12%	12%	12%	12%	12%	12%	12%	12%
F4.5	Seeds	28%	27%	27%	27%	26%	26%	26%	26%	26%	26%	26%	26%	26%	26%	26%	26%	26%	26%	26%	26%	26%	26%
F4.6	Mineral fertilizers to agriculture	11%	11%	11%	11%	11%	11%	11%	11%	11%	11%	11%	11%	11%	11%	11%	11%	11%	11%	11%	11%	11%	11%
F4.7	Export feed	23%	23%	23%	23%	24%	12%	12%	12%	12%	12%	12%	12%	12%	12%	12%	12%	12%	12%	12%	12%	12%	12%
F4.8	Export food	23%	21%	20%	19%	13%	13%	13%	13%	13%	13%	13%	13%	13%	12%	12%	11%	11%	11%	11%	10%	10%	10%
F4.9	Export mineral fertilizers	14%	14%	14%	14%	14%	14%	14%	14%	14%	14%	14%	14%	14%	14%	14%	14%	14%	14%	14%	14%	14%	14%
F4.10	Export chemicals	22%	20%	18%	17%	15%	12%	12%	12%	12%	12%	12%	12%	12%	12%	12%	12%	12%	12%	12%	12%	12%	12%
F4.11	Municipal ind. WW	21%	22%	22%	22%	23%	23%	24%	25%	21%	20%	20%	19%	19%	19%	18%	16%	16%	17%	17%	18%	19%	20%
F4.12	Detergents	23%	22%	22%	22%	21%	21%	21%	21%	20%	20%	9%	8%	8%	7%	4%	7%	8%	8%	9%	10%	12%	13%
F4.13	Min. fertilizers to consumers	25%	25%	25%	25%	25%	25%	25%	25%	25%	25%	22%	20%	18%	17%	16%	15%	14%	13%	13%	12%	11%	12%
F4.14	Pet food	64%	64%	64%	64%	64%	64%	63%	63%	62%	62%	61%	61%	60%	59%	59%	58%	57%	57%	57%	57%	57%	57%
F4.15	Food	20%	20%	20%	20%	20%	17%	17%	17%	17%	17%	17%	17%	17%	16%	16%	16%	15%	15%	15%	15%	15%	15%
F4.16	Other industrial waste	36%	36%	36%	36%	36%	36%	34%	32%	29%	27%	26%	26%	26%	26%	26%	26%	26%	26%	26%	26%	26%	26%
F4.17	Vegetal industrial waste	43%	42%	43%	43%	42%	44%	45%	42%	40%	39%	38%	37%	36%	36%	36%	36%	36%	36%	36%	36%	36%	36%
F4.18	Animal industrial waste	69%	69%	69%	69%	64%	60%	56%	52%	49%	46%	44%	43%	43%	42%	41%	41%	41%	42%	42%	41%	42%	43%
F4.19	Marketable feed	58%	52%	47%	43%	39%	37%	35%	34%	32%	32%	31%	19%	19%	19%	19%	19%	19%	19%	19%	19%	19%	31%
F4.20	In situ ind. WW	10%	9%	8%	8%	7%	4%	7%	8%	8%	9%	10%	12%	13%	11%	10%	10%	10%	10%	10%	11%	12%	12%

Flow N.	Flow name	1990	1991	1992	1993	1994	1995	1996	1997	1998	1999	2000	2001	2002	2003	2004	2005	2006	2007	2008	2009	2010	2011
F5.1	Biomass ashes to landscaping	56%	56%	56%	56%	50%	44%	40%	37%	34%	32%	30%	29%	28%	27%	25%	27%	25%	27%	25%	27%	25%	27%
F5.2	Biomass ashes to fields	56%	56%	56%	56%	50%	44%	40%	37%	34%	32%	30%	29%	28%	27%	25%	27%	25%	27%	25%	27%	25%	27%
F5.3	Biomass ashes to green areas	56%	56%	56%	56%	50%	44%	40%	37%	34%	32%	30%	29%	28%	27%	25%	27%	25%	27%	25%	27%	25%	27%
F5.4	Biogas digestates																						
F5.5	Landfilled biomass ashes	52%	52%	52%	52%	44%	38%	33%	29%	25%	23%	20%	18%	17%	16%	12%	12%	12%	12%	12%	12%	12%	16%
F5.6	Biofuels by-products as feed	15%	15%	15%	15%	15%	15%	15%	15%	15%	15%	15%	15%	23%	52%	52%	52%	52%	52%	52%	52%	52%	52%
F5.7	Import raw materials for biofuels	12%	12%	12%	12%	12%	12%	12%	12%	12%	12%	12%	12%	21%	60%	60%	60%	60%	60%	60%	60%	60%	60%
F6.1	Municipal households WW	21%	22%	22%	22%	23%	23%	24%	25%	21%	20%	20%	19%	19%	19%	18%	16%	16%	17%	17%	18%	19%	20%
F6.2	Residual waste	33%	33%	33%	33%	33%	33%	27%	29%	29%	30%	34%	32%	31%	29%	28%	28%	25%	28%	28%	29%	31%	32%
F6.3	Separate org. waste	27%	27%	27%	27%	27%	27%	27%	27%	27%	27%	27%	27%	29%	29%	27%	29%	27%	29%	27%	26%	28%	29%
F6.4	Green waste	52%	53%	52%	53%	54%	27%	27%	29%	30%	31%	32%	31%	30%	29%	27%	29%	30%	29%	27%	27%	29%	29%
F6.5	Waste wood and paper	36%	36%	36%	36%	36%	36%	36%	33%	31%	29%	26%	26%	28%	28%	26%	28%	26%	28%	26%	26%	28%	28%
F6.6	Fecal sludge to groundwater	51%	51%	50%	51%	51%	51%	51%	51%	51%	52%	52%	53%	54%	55%	55%	55%	55%	55%	55%	55%	55%	55%
F6.7	Fecal sludge to agriculture	51%	51%	50%	51%	51%	51%	51%	51%	51%	52%	52%	53%	54%	55%	55%	55%	55%	55%	55%	55%	55%	55%
F7.1	Sewage sludge																						
F7.2	WW effluents																						
F7.3	Stormwater overflow	56%	56%	49%	44%	39%	35%	33%	31%	29%	28%	27%	26%	27%	27%	27%	28%	29%	31%	33%	36%	39%	44%
F8.1	Compost to consumers	26%	24%	22%	21%	20%	19%	19%	18%	18%	18%	17%	18%	18%	18%	19%	20%	21%	22%	24%	26%	28%	28%
F8.2	M&B meal to animal feed	33%	32%	30%	30%	29%	28%	28%	28%	28%	27%	27%	12%	12%	12%	12%	12%	12%	12%	12%	12%	12%	12%
F8.3	Substrate landscaping																						
F8.4	Recycled wood and paper																						
F8.5	Wastes recycled in agriculture																						
F8.6	Export sewage sludge	59%	58%	51%	45%	40%	35%	34%	32%	29%	29%	28%	26%	27%	25%	25%	24%	24%	24%	27%	27%	27%	29%
F8.7	Export M&B meal	56%	56%	56%	56%	50%	45%	40%	37%	34%	32%	31%	30%	29%	28%	26%	26%	26%	28%	29%	30%	31%	32%
F8.8	Export filter cakes																						
F8.9	Export organic waste	83%	83%	83%	83%	83%	83%	83%	83%	83%	83%	83%	83%	83%	83%	47%	47%	47%	83%	83%	47%	83%	83%
F8.10	Waste to biogas plants																						
F8.11	Green waste to biomass plants	81%	81%	81%	81%	81%	81%	81%	81%	81%	81%	81%	81%	81%	81%	81%	81%	81%	81%	81%	81%	81%	81%
F8.12	Import animal waste	60%	60%	60%	60%	54%	49%	45%	42%	40%	38%	37%	36%	35%	35%	33%	33%	33%	35%	35%	36%	37%	38%
F9.1	Import water bodies	30%	30%	30%	30%	30%	30%	30%	30%	29%	29%	29%	29%	29%	29%	29%	29%	29%	29%	29%	29%	29%	29%
F9.2	Export water bodies	23%	23%	23%	23%	23%	23%	23%	23%	23%	23%	23%	23%	23%	23%	23%	23%	23%	23%	23%	23%	23%	23%
P1.1	Meat production	22%	20%	19%	18%	15%	15%	15%	15%	15%	15%	15%	15%	15%	14%	14%	13%	13%	13%	13%	12%	12%	12%
P1.2	Eggs and milk production	13%	13%	13%	13%	13%	13%	13%	13%	13%	13%	13%	13%	13%	13%	12%	12%	12%	11%	11%	11%	11%	11%
P3.1	Timber	31%	31%	29%	29%	29%	29%	29%	31%	31%	31%	29%	29%	29%	31%	31%	31%	31%	29%	29%	29%	31%	31%
P4.1	Products to food production																						
P4.2	Products to feed production																						
P4.3	By-products to animal feed production	61%	55%	50%	46%	43%	41%	39%	38%	37%	36%	36%	26%	26%	26%	26%	26%	26%	26%	26%	26%	26%	36%
P4.4	Food WW	56%	56%	56%	56%	56%	56%	56%	56%	56%	56%	56%	56%	56%	56%	56%	56%	56%	56%	56%	56%	56%	56%
P4.5	Animal feed WW	56%	56%	56%	56%	56%	56%	56%	56%	56%	56%	56%	56%	56%	56%	56%	56%	56%	56%	56%	56%	56%	56%
P4.6	Fertilizer WW	80%	80%	80%	80%	80%	80%	80%	80%	80%	80%	80%	80%	80%	80%	80%	80%	80%	80%	80%	80%	80%	80%
P4.7	Chemical WW	80%	80%	80%	80%	80%	80%	80%	80%	80%	80%	80%	80%	80%	80%	80%	80%	80%	80%	80%	80%	80%	80%
P4.8	Fertilizer industry in situ WW	40%	37%	35%	32%	30%	28%	30%	32%	35%	37%	40%	43%	43%	43%	43%	43%	43%	43%	43%	43%	43%	43%
P4.9	Chemical industry in situ WW	40%	37%	35%	32%	30%	28%	30%	32%	35%	37%	40%	43%	43%	43%	43%	43%	43%	43%	43%	43%	43%	43%

Flow N.	Flow name	1990	1991	1992	1993	1994	1995	1996	1997	1998	1999	2000	2001	2002	2003	2004	2005	2006	2007	2008	2009	2010	2011
P6.1	Organic waste to home composting	36%	34%	32%	31%	30%	30%	28%	30%	30%	28%	30%	30%	30%	30%	28%	30%	28%	30%	30%	28%	30%	30%
P6.2	Garden vegetables	20%	19%	19%	19%	18%	18%	18%	18%	18%	18%	18%	18%	18%	18%	18%	18%	18%	18%	18%	18%	18%	18%
P6.3	Garden waste	35%	36%	35%	36%	37%	35%	35%	36%	37%	38%	37%	36%	34%	33%	31%	32%	32%	31%	29%	29%	30%	30%
P6.4	Domestic animals excretions	80%	80%	80%	80%	80%	80%	80%	80%	80%	80%	80%	80%	80%	80%	80%	80%	80%	80%	80%	80%	80%	80%
P6.5	Wood & paper in residual waste	39%	39%	38%	38%	38%	37%	34%	32%	30%	28%	27%	27%	27%	27%	26%	27%	27%	27%	26%	26%	27%	27%
P7.1	Municipal WW to MWWTP	23%	20%	18%	17%	16%	8%	13%	13%	8%	13%	13%	8%	10%	11%	10%	10%	8%	10%	10%	10%	8%	8%
P7.2	Municipal effluent	21%	18%	16%	14%	13%	8%	13%	13%	8%	13%	13%	8%	10%	11%	10%	10%	8%	10%	10%	10%	8%	8%
P7.3	Municipal sewage sludge	23%	20%	18%	17%	16%	8%	13%	13%	8%	13%	13%	8%	10%	11%	10%	10%	8%	10%	10%	10%	8%	10%
P7.4	Industrial effluent	10%	9%	8%	8%	7%	4%	7%	8%	8%	9%	10%	12%	13%	15%	17%	20%	22%	22%	22%	22%	22%	22%
P7.5	Industrial sewage sludge	10%	9%	8%	8%	7%	4%	7%	8%	8%	9%	10%	12%	13%	15%	17%	20%	22%	22%	22%	22%	22%	22%
P8.1	Animal waste to Rendering	29%	29%	28%	28%	27%	27%	27%	27%	27%	27%	27%	27%	27%	27%	27%	27%	27%	27%	27%	27%	27%	27%
P8.2	Green waste to compost	40%	37%	34%	32%	31%	30%	29%	28%	26%	26%	26%	28%	29%	30%	31%	32%	34%	37%	40%	45%	50%	56%
P8.3	Animal waste to biogas	39%	39%	39%	39%	38%	37%	36%	35%	35%	34%	34%	34%	33%	33%	33%	33%	33%	33%	33%	33%	33%	33%
P8.4	HH res. waste to MBT	47%	47%	47%	47%	47%	47%	40%	40%	39%	36%	37%	36%	36%	36%	34%	34%	40%	34%	34%	34%	35%	36%
P8.5	HH res. waste to landfill	47%	47%	47%	47%	47%	47%	40%	40%	39%	36%	37%	36%	36%	36%	34%	34%	40%	34%	34%	34%	35%	36%
P8.6	HH res. waste to TT	47%	47%	47%	47%	47%	47%	40%	40%	39%	36%	37%	36%	36%	36%	34%	34%	40%	34%	34%	34%	35%	36%
P8.7	SS to composting	31%	29%	28%	27%	27%	25%	27%	26%	24%	26%	26%	24%	25%	26%	26%	26%	25%	25%	25%	24%	24%	25%
P8.8	SS to landfill	24%	21%	20%	18%	17%	13%	17%	17%	13%	16%	16%	13%	14%	15%	14%	15%	13%	15%	14%	4%	4%	4%
P8.9	SS to TT	24%	21%	20%	18%	17%	13%	17%	17%	15%	17%	17%	13%	14%	18%	17%	18%	16%	18%	18%	17%	17%	18%
P8.10	SS to co-incineration	30%	28%	25%	22%	19%	15%	17%	16%	12%	15%	14%	10%	10%	11%	10%	10%	8%	10%	12%	12%	11%	13%
P8.11	SS applied in agriculture	24%	21%	20%	18%	17%	13%	17%	17%	13%	16%	16%	13%	14%	15%	14%	15%	13%	15%	14%	14%	13%	15%
P8.12	SS to landscaping	32%	29%	29%	29%	29%	29%	32%	34%	35%	40%	45%	49%	56%	56%	56%	56%	55%	56%	56%	56%	55%	56%
P8.13	M&B meal as fertilizer	41%	41%	41%	41%	41%	41%	41%	41%	41%	41%	41%	32%	30%	30%	29%	28%	28%	28%	28%	28%	28%	28%
P8.14	M&B meal to fuel	41%	41%	41%	41%	41%	41%	41%	41%	41%	41%	57%	34%	34%	34%	27%	27%	27%	27%	27%	27%	27%	28%
P8.15	Stabilized waste to landfill	76%	76%	76%	71%	68%	65%	63%	61%	60%	59%	59%	58%	58%	57%	57%	57%	58%	58%	59%	59%	60%	61%
P8.16	Compost to agriculture	26%	24%	22%	21%	20%	19%	19%	18%	18%	18%	17%	18%	18%	18%	19%	20%	21%	22%	24%	26%	28%	28%
P8.17	Compost to landscaping	26%	24%	22%	21%	20%	19%	19%	18%	18%	18%	17%	18%	18%	18%	19%	20%	21%	22%	24%	26%	28%	28%
P8.18	Stabilized waste to TT	58%	58%	58%	52%	47%	43%	40%	37%	35%	34%	33%	32%	32%	30%	30%	30%	32%	32%	33%	34%	35%	37%
P8.19	Ash co-inc. to landfill																						
P8.20	Ash/slag to landfill																						
P8.21	Loss in clinker																						
P8.22	MSS to fuel	32%	30%	29%	28%	27%	25%	27%	27%	23%	23%	22%	18%	18%	17%	16%	16%	14%	15%	14%	15%	14%	16%
P8.23	MSS to waste incineration	32%	30%	29%	28%	27%	25%	27%	27%	23%	23%	22%	18%	18%	17%	16%	16%	14%	15%	14%	15%	14%	16%
P8.24	Filter cakes Inc.																						
P8.25	Filter cakes Co-inc.																						
P8.26	SS to MBT	59%	58%	57%	50%	45%	39%	37%	34%	30%	30%	29%	27%	27%	25%	25%	24%	26%	27%	28%	29%	30%	33%
P8.27	Animal waste to compost	39%	39%	39%	39%	38%	37%	36%	35%	35%	34%	34%	34%	33%	33%	33%	33%	33%	33%	33%	33%	33%	33%
P8.28	Veg. ind. to biogas	24%	24%	24%	24%	24%	24%	24%	24%	24%	24%	24%	24%	24%	31%	42%	59%	59%	59%	59%	59%	59%	59%

Table S3-2: Relative uncertainty of the input data for the stocks, stock change rates and transfer coefficients.

Empty lines indicate that no input values were calculated and that they were calculated directly by the software STAN, according to the principle of mass conservation.

Stock N.	Stock name	1990	1991	1992	1993	1994	1995	1996	1997	1998	1999	2000	2001	2002	2003	2004	2005	2006	2007	2008	2009	2010	2011
S1	Livestock	14.5%																					
S2	Agricultural fields	51.0%																					
S3	Trees and soil	50.6%																					
S6-1	Stock in buildings and furniture																						
S6-2	Private gardens & public green areas	60.4%																					
S8-1	Clinker	39.9%																					
S8-2	Landfills	70.3%																					
S9	Water bodies																						

Stock N.	Stock change rate name	1990	1991	1992	1993	1994	1995	1996	1997	1998	1999	2000	2001	2002	2003	2004	2005	2006	2007	2008	2009	2010	2011
S1	Livestock	15%	15%	15%	15%	15%	15%	15%	15%	15%	15%	15%	15%	15%	15%	15%	15%	15%	15%	15%	15%	15%	15%
S2	Agricultural fields																						
S3	Trees and soil																						
S6-1	Stock in buildings and furniture																						
S6-2	Private gardens & public green areas																						
S8-1	Clinker																						
S8-2	Landfills																						
S9	Water bodies																						

Process N.	Process name (transfer coefficient)	1990	1991	1992	1993	1994	1995	1996	1997	1998	1999	2000	2001	2002	2003	2004	2005	2006	2007	2008	2009	2010	2011
P8.17	Waste incineration	9%	9%	8%	8%	7%	6%	6%	6%	5%	5%	5%	5%	4%	4%	4%	5%	5%	5%	5%	6%	6%	6%
P8.18	Sludge co-incineration	9%	9%	9%	9%	9%	9%	9%	9%	9%	8%	8%	7%	6%	6%	6%	5%	5%	5%	5%	4%	5%	5%
