# Supplementary material for: Added Values of Time Series in Material Flow Analysis: The Austrian Phosphorus Budget from 1990 to 2011
Source: J Ind Ecol. 2015 Dec 22;20(6):1334–48. doi: 10.1111/jiec.12381 (PMC5217078; doi:10.1111/jiec.12381)
Supplement: Supplementary file 4 — Supporting Information S4: This supporting information displays the degree of impact that reconciliation has had on the whole system. [file 44498_2016_2006008_MOESM4_ESM.pdf]

## SUPPORTING INFORMATION FOR:

Zoboli, O., D. Laner, M. Zessner, and H. Rechberger. 2015. Added values of time series in MFA: The Austrian phosphorus budget from 1990 to 2011. *Journal of Industrial Ecology.*

**Summary**

This supporting information displays the degree of impact that reconciliation has had on the whole system.

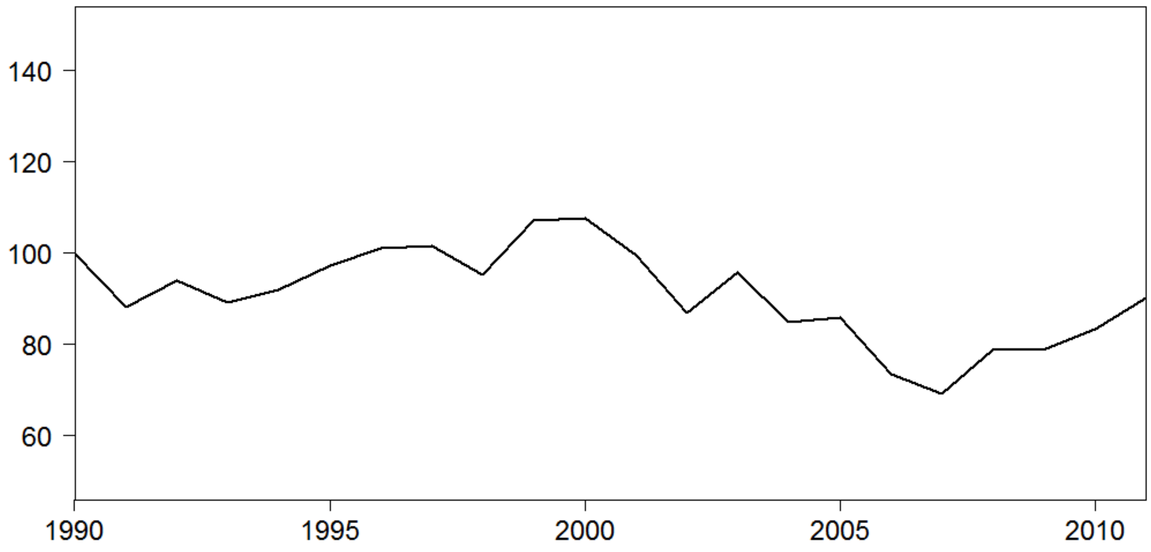


**Figure S4-1: Degree of the impact of reconciliation on the whole system from 1990 to 2011, normalized with respect to 1990**
